# Evolution and Spread of Regionally Adapted Newcastle Disease Virus Isolates From Live Bird Markets in Nigeria, 2023–2024

**DOI:** 10.1155/tbed/8829822

**Published:** 2026-01-24

**Authors:** Mohammed Usman Sajo, Dongyeop Lee, Jean Nepomuscene Hakizimana, Augustino Chengula, Abdul-Dahiru El-Yuguda, Dong-Hun Lee, Gerald Misinzo

**Affiliations:** ^1^ Department of Microbiology, Parasitology and Biotechnology, College of Veterinary Medicine and Biomedical Sciences, Sokoine University of Agriculture, Morogoro, 67152, Tanzania, suanet.ac.tz; ^2^ OR Tambo Africa Research Chair for Viral Epidemics, SACIDS Foundation for One Health, Sokoine University of Agriculture, Morogoro, 67152, Tanzania, suanet.ac.tz; ^3^ Animal Virus Research Laboratory, Department of Veterinary Microbiology, Faculty of Veterinary Medicine, University of Maiduguri, Maiduguri, 600001, Nigeria, unimaid.edu.ng; ^4^ Konkuk University Zoonotic Disease Research Center, College of Veterinary Medicine, Konkuk University, Seoul, 05029, Republic of Korea, konkuk.ac.kr; ^5^ Wildlife Health Laboratory, College of Veterinary Medicine, Konkuk University, Seoul, 05029, Republic of Korea, konkuk.ac.kr

**Keywords:** evolution, food security, molecular epidemiology, Newcastle disease virus, Nigeria

## Abstract

Newcastle disease (ND) virus (NDV) infection ranks among the most important poultry diseases globally. In Nigeria, ND remains a persistent menace to poultry production, marked by recurrent outbreaks. However, there is limited understanding of the evolutionary changes and transmission dynamics of the virus in the region. A molecular epidemiological study was conducted to elucidate the evolutionary and transmission patterns of NDV in Nigeria. Phylogenetic analysis of seven NDV isolates from cases recorded between 2023 and 2024 in four Northeastern states exhibited genetic diversity and formed distinct clusters that correspond to the prevailing subgenotype XIV.2. The maximum clade credibility (MCC) tree suggests sustained local circulation of the dominant NDV lineage, likely preceded by an international introduction from Southeast Asia. The fusion genes of the Nigerian genotype XIV and another important genotype XVII are mainly under negative selection, but codons 516 (XIV) and 114 (XVII) consistently show positive selection. The Nextstrain analysis reveals ongoing local evolution and genetic diversity of NDV in West Africa, and Central Nigeria acting as a key transmission hub, with evidence of reintroductions from neighboring countries. These findings have implications for NDV control and prevention strategies in Nigeria, highlighting the need for enhanced NDV surveillance, transboundary transmission control, and development of a vaccine tailored to the circulating NDV genotypes. The study also contributes to the understanding of regional spread pattern of NDV and informs evidence‐based policies for mitigating the impact of the disease on poultry production.

## 1. Introduction

Newcastle disease (ND) is one of the notifiable poultry diseases worldwide due to its high transmissibility, severe economic impact, and importance in international trade regulations [1]. The disease is highly contagious and fatal, affecting poultry and a wide range of wild birds [2]. The epizootics of ND continue to pose a challenge in Africa, often receiving limited attention [3], while countries like the United States and China have effectively controlled virulent ND outbreaks [4, 5] and interestingly, France has yet to report an outbreak of the disease in chickens, with cases confined to other poultry species [6, 7]. ND is caused by avian paramyxovirus type 1 (APMV‐1) also known as ND virus (NDV; species, *Orthoavulavirus javaense*), which is an enveloped virus containing a linear, non‐segmented, single‐stranded, negative‐sense RNA genome [8] consisting of 15,186 – 15,198 nucleotides [9, 10]. It contains six genes that encode various proteins comprising nucleocapsid protein (NP), phosphoprotein (P), matrix protein (M), fusion protein (F), hemagglutinin‐neuraminidase protein (HN), and large polymerase protein (L) [11]. Additionally, during the transcription of the P gene, two nonstructural proteins (V and W proteins) are produced [12]. The HN and F proteins are located on the surface of the virus membrane, while the NP, P, and L proteins form the genetic material complex of the virus. Phylogenetic studies at the molecular level of the NDV fusion protein precusor (F0) cleavage site have determined the consensus amino acid sequence ^112^R/K‐R‐Q‐R/K‐R‐F^117^ for virulent strains and ^112^G/E‐K/R‐Q‐G/E‐R‐L^117^ for NDV strains of low virulence [13].

According to Dimitrov et al. [14], using phylogenetic analysis, NDV can be categorized into two separate classes, namely class 1 and class 2. Class 1 consists of a single genotype (with three subgenotypes: 1.1.1, 1.1.2, and 1.2), while class II encompasses a total of at least 20 genotypes, identified as 2.I to 2.XXI. Genotype XV within class II, which contains only recombinant sequences, was later excluded. Subgenotypes, initially labeled with letters (e.g., XIVb) [15], were later reclassified using Arabic numerals (e.g., XIV.2) in the updated system [14]. It is shown that in North African countries, such as Egypt, NDV outbreaks are primarily caused by genotypes 2.II, 2.VI, and 2.VII. Eastern African countries, like Tanzania, have circulating strains belonging to genotypes 2.V, 2.VII, and 2.XIII [16, 17]. Countries like Nigeria, Niger, and other West African countries (Mali, Mauritania, Ivory Coast, and Burkina Faso) have newly circulating genotypes (2.XIV, 2.XVII, and 2.XVIII) that are isolated and limited to this region, along with other genotypes such as II, VII, and V [17]. In Southern African countries (South Africa, Madagascar, and Mozambique), prevalent genotypes include 2.II, 2.VII, 2.VIII, 2.XI, and 2.XIII [16, 17]. Genotypes 2.XIV, 2.XVII, and 2.XVIII have been exclusively reported in West and Central Africa, with subgenotype 2.XVII.2 documented only in Nigeria [18]. The tropical climate of Nigeria, characterized by fluctuating temperatures, humidity and rainfall, supports the persistence and spread of ND in poultry flocks [19]. Studies have shown that the endemicity and high genetic diversity of NDV in Nigeria could be a result of previous spillover of NDV from wild birds to domestic chickens as well as the evolution of the region‐specific virus strains [18]. Subgenotype 2.XIV.2 has been isolated in a commercial farm in Nigeria that has vaccinated with an unspecified vaccine strain against NDV [16] and recently, the same velogenic subgenotype 2.XIV.2 was isolated among a flock vaccinated with LaSota and Komarov ND vaccine strains following an outbreak in Kano State, Nigeria [20]. Funsho‐Sanni and colleagues asserted that A2 antigenic epitope plays a role in the induction of antibody escape mutation among the said subgenotype, and they concluded that in order to generate genetically matched vaccines in Nigeria, ND surveillance and molecular analysis of circulating strains should be encouraged and reported. Despite several seroprevalence and pathological studies of NDV in Nigeria, limited data are available on the sequence and spatial phylogenetics of NDV in the country, especially the Northeastern region that holds a critical geographical position for cross‐border disease transmission [18]. This study aimed to identify the existing or emerging NDV strains circulating in Northeastern Nigeria and to analyze their evolutionary patterns to better understand the virus’s spread and support targeted control strategies.

## 2. Materials and Methods

### 2.1. Study Area

The Northeast zone of Nigeria, which comprises about one fourth of the country’s land mass, is situated within 9°–14°N and 8°–15°E [21]. A considerable proportion of Nigeria’s 208 million poultry birds [22, 23], ~33 million heads or about 16% [24], are found in the Northeastern region. Politically, the zone comprises Adamawa, Bauchi, Borno, Gombe, Taraba, and Yobe States. Borno State shares international boundaries with Cameroon, Chad, and Niger Republic, while Adamawa, Taraba, and Yobe States share international boundaries with Cameroon and Niger Republic, respectively. The Northeast region holds a critical geographical position, bordering three countries from the west, north‐central, and central parts of Africa, making it susceptible to transboundary disease transmission. The monthly mean temperature in the region can reach up to 40.6°C, particularly in the extreme north. Wild birds, including species that migrate seasonally between Africa and Europe, are also abundant in the region. Environmental scarcity caused by low rainfall has affected agricultural activity, especially animal husbandry, leading to human conflicts [25–27].

### 2.2. Sampling Procedure

One oropharyngeal swab and six pooled organ tissue samples were purposively collected from one clinically sick and six dead village chickens, respectively, in live bird markets (LBMs) within the four Northeastern Nigerian states (Borno, Gombe, Taraba, and Yobe) from 22 December 2023 to 18 May 2024 (Table 1). The extended timeline reflects the regular occurrence of NDV cases in village chickens within the region. The pooled organ tissue samples per bird included the proventriculus, spleen, intestine, liver, lungs, and trachea, collected opportunistically from freshly dead bird carcasses with visible presumptive pathological lesions of NDV. Samples were collected in single‐use viral transport tubes each containing penicillin (10,000 units/mL), streptomycin (10,000 mg/mL), gentamicin (5000 mg/mL), and amphotericin B (50 mg/mL) with 50% glycerol adjusted to pH 7.2 and transported to the National Veterinary Research Institute (NVRI), Vom, under cold chain conditions.

**Table 1:** Details of NDV isolates described in this study.

Accession number	Isolate	State	Location	Coordinates N, E	Sample type	Collection date	Cleavage site
PX095534	SJ55	Gombe	LBM Tashan Dukku	10.301150, 11.159706	OP swab	22‐Dec‐23	RRRKRF
PX095535	SJ106	Taraba	Main LBM Jalingo	8.894608, 11.359054	Tissues	24‐Jan‐24	RRRKRF
PX095537	SJ117	Borno	LBM Monday Market	11.835608, 13.152259	Tissues	15‐May‐24	RRRKRF
PX095537	SJ124	Borno	LBM Monday Market	11.835608, 13.152259	Tissues	15‐May‐24	RRRKRF
PX095538	SJ126	Yobe	LBM Damaturu	11.7382305, 11.9322155	Tissues	18‐May‐24	RRRKRF
PX095539	SJ129	Yobe	LBM Damaturu	11.7382305, 11.9322155	Tissues	18‐May‐24	RRRKRF
PX095540	SJ130	Yobe	LBM Damaturu	11.7382305, 11.9322155	Tissues	18‐May‐24	RRRKRF

*Note:* Tissues: multiple organ tissues.

Abbreviations: LBM, live birds market; OP, oropharyngeal.

### 2.3. Virus Isolation

The samples of about 1.5 mL each were filtered through a 0.22 μm PES syringe filter (Microlab Scientific Co., Ltd, Shanghai, China). Following filtration, 0.2 mL of the resulting filtrate was inoculated into 10‐day‐old specific‐antibody‐negative (SAN) embryonated chicken eggs for blind passage (3 eggs per sample), incubating them at 37°C for 3–5 days according to a previously established protocol [13, 28]. The hemagglutination (HA) test was conducted on the allantoic fluids with the isolated viral strains using 1% suspension of chicken red blood cells, following established protocols [29]. The details of the sampled isolates in this study are provided in Table 1.

### 2.4. Nucleic Acid Extraction, Amplification, and Sequencing

The HA positive allantoic fluids were centrifuged at 3000 rpm for 15 min, filtered through 0.45‐μm syringe filters, and treated with DNase I (TransGen Biotech, Beijing, China) to reduce the chances of host genome sequences. Viral RNA extraction was performed with QIAGEN RNeasy Mini Kit (QIAgen, Hilden, Germany) following the manufacturer’s guide. The RNAs were screened for positive NDV using reverse transcription polymerase chain reaction (RT‐PCR) targeting the partial F gene, adhering to the established procedure [30]. The RNAs were treated with 0.5 μL of DNase I (1 U/μL) (Thermo Fisher Scientific, MA, USA) per reaction mixture containing 5 μL of 10 × reaction buffer, and incubated at 37°C for 45 min before inactivating the enzyme with 5 μL EDTA at 65°C for 10 min. As described previously [31], single‐stranded cDNA (sscDNA) was synthesized in a 20‐μL reaction mixture containing 6 μL of nuclease‐free water, 5 μL of viral nucleic acids, 1 μL of 10‐μM dNTPs, 1 μL of 100‐pmol primer K‐8N (GACCATCTAGCGACCTCCACNNNNNNNN), 1 μL of 100‐mM dithiothreitol (DTT), 4 μL of SS IV buffer, 1 μL of RNase inhibitor, and 1 μL of SuperScript IV Reverse Transcriptase (Thermo Fisher Scientific, MA, USA), following the manufacturer’s instructions. The sscDNA was converted into double‐stranded cDNA (dscDNA) by heating the entire 20‐μL sscDNA reaction to 95°C for 3 min and then cooled to 4°C for 5 min after mixing with 0.5 μL of 10‐pmol of primer K‐8N and 0.5 μL of 10‐μM dNTPs and 3 μL of 1 × Klenow reaction buffer (New England Biolabs, MA, USA). Subsequently, 5 μL of nuclease‐free water and 1 μL of Klenow fragment (enzyme) were added to the reaction and then incubated at 37°C for 60 min, followed by enzyme inactivation at 75°C for 15 min in a final volume of 30 μL. The dsDNA was then purified using QIAquick PCR Purification Kit (QIAGEN, Hilden, Germany). A sequence‐independent PCR amplification was performed using 5 μL of the purified dsDNA template in a 50‐μL total reaction mixture containing 17.5 μL nuclease‐free water, 2.5 μL of 10‐μM K‐primer (GACCATCTAGCGACCTCCAC), and 25 μL Phusion HF PCR master mix (New England Biolabs, MA, USA). The PCR cycling conditions consisted of an initial denaturation at 98°C for 30 s, followed by 35 cycles of 98°C for 10 s, 55°C for 30 s, and 72°C for 1 min, with a final extension at 72°C for 10 min. The PCR product was visualized by gel electrophoresis using 1% agarose LE (iNtRON Biotechnology, Gyeonggi‐do, Republic of Korea). The final PCR product was purified using Agencourt AMPure XP beads (Beckman Coulter, CA, USA). The purified dsDNA concentration was quantified using the Qubit 1x dsDNA HS assay (Invitrogen, MA, USA) according to the manufacturer’s instructions. The quantified dscDNA was prepared for next generation sequencing at the Wildlife Laboratory of Konkuk University, Republic of Korea. The sequencing library was prepared from the synthesized cDNA using the Illumina DNA Prep kit (Illumina, CA, USA). All libraries underwent equimolar dilution and were pooled. The library pool was loaded into the 300‐cycle MiniSeq High Output Reagent Kit (Illumina, CA, USA), and paired‐end sequencing (2 × 150 bp) was performed on the Illumina MiniSeq instrument (Illumina, CA, USA). The process from RNA extraction to sequencing was adapted from Lee [32].

### 2.5. Sequence Data Assembly

Raw Illumina paired‐end reads were filtered with BBDuk (v38.84) to trim adapters and remove low‐quality bases (> Q20) from both ends. Geneious Assembler (version 2025.0.3) and metaSPAdes (v4.0.0) were used for *de novo* assembly. Closely related NDV reference genomes (GenBank Accession numbers MH996989 and PV137995) obtained from contigs BLAST were used for the reference‐guided assembly. Due to insufficient coverage, likely caused by RNA degradation from prolonged transport, only consensus sequences of the full‐length F and HN genes were extracted from the assemblies of the seven samples, with a minimum base agreement threshold of 50%.

### 2.6. Phylogenetic Analyses

Seven complete F gene sequences were analyzed by BLAST query of the NCBI Virus database to retrieve the top 500 hits as of April 27, 2025. These sequences were aligned using MAFFT (v7.490), trimmed to match the F gene region, and filtered for redundancy with a 100% identity threshold using ElimDupes software (https://www.hiv.lanl.gov/content/sequence/elimdupesv2/elimdupes.html), resulting in a total of 286 distinct sequences (Table S1). Maximum likelihood (ML) phylogenies were constructed using RAxML (v8.2.12), applying the general time reversible (GTR) model with a gamma distribution and 1000 bootstrap replicates [33]. Likewise, ML tree was constructed using the seven sampled and 421 retrieved HN gene sequences following the same procedure applied for the F gene sequences. The ML trees were visualized using iTOL version 6 [34]. TempEst v1.5.3 was used to assess the temporal signal and root‐to‐tip divergence of the dataset (Figure S1), supporting the use of molecular clock models in subsequent Bayesian evolutionary analysis sampling trees (BEAST) analyses.

ModelFinder in IQ‑TREE [35] was used to select the best‐fitting nucleotide substitution model, and the GTR substitution model with gamma + invariant site heterogeneity model was chosen for downstream Bayesian phylogenetic analysis in BEAST, utilizing an uncorrelated lognormal relaxed clock. The Bayesian phylogenetic tree of 286 F gene sequences was reconstructed using BEAST v.1.10.4 (http://beast.bio.ed.ac.uk). The tree prior was specified as the Gaussian Markov random field (GMRF) Bayesian Skyride model. This model was chosen for its robustness in capturing smooth population changes, particularly under sparse or uneven sequence sampling [36]. A chain length of 50 million generations was used, discarding the first 10% as burn‐in. Only parameters with effective sample size (ESS) > 200 were retained for reliable inference. Maximum clade credibility (MCC) tree was generated using Tree Annotator v1.10.4. The MCC tree was then displayed using the visualization tool (Figtree v1.4.4).

### 2.7. Selection Pressure Analysis

Selection pressure analysis was performed by estimating dN/dS (*ω*) ratios using online Datamonkey web server (https://www.datamonkey.org/) to assess diversifying and purifying selection across coding sequences within the HyPhy Vision (https://vision.hyphy.org/). The F gene sequences of the Nigerian class II genotype XIV strains (*n* = 57) and another important genotype XVII strains (*n* = 54), obtained through the BLAST hit of isolates from this study, were analyzed. The average *ω* values were estimated using the fixed effects likelihood (FEL), mixed effects model of evolution (MEME) methods, and single‐likelihood ancestor counting (SLAC), applying a *p*‐value threshold of 0.1, while Fast Unconstrained Bayesian AppRoximation (FUBAR) results were considered significant with a posterior probability above 0.9. Likelihood ratio tests were applied to determine the statistical significance of selection, with results visualized to interpret functional impacts on protein structure.

### 2.8. Nextstrain Phylogeographic Analysis

Transmission mapping of West African NDV F gene sequences from this study (Table 2 and Table S2) was conducted using Nextstrain, an open‐source platform for real‐time tracking of pathogen evolution [37]. The resulting province‐specific builds were visualized using Nextstrain’s Auspice platform to reveal the phylogeographic patterns [37].

**Table 2:** The summary of isolates per state used for the Nextstrain analysis.

Country	Location	Sequence number
Nigeria	Plateau	14
Nigeria	Abuja	5
Nigeria	Kogi	1
Nigeria	Bauchi	5
Nigeria	Kano	12
Nigeria	Kaduna	6
Nigeria	Katsina	7
Nigeria	Jigawa	7
Nigeria	Sokoto	1
Nigeria	Nasarawa	3
Nigeria	Borno	4
Nigeria	Yobe	6
Nigeria	Adamawa	1
Nigeria	Gombe	4
Nigeria	Taraba	3
Nigeria	Imo	2
Nigeria	Ogun	1
Nigeria	Kwara	1
Nigeria	Kebbi	1
Nigeria	Oyo	1
Nigeria	Rivers	1
Nigeria	Zamfara	1
Niger	Niamey	5
Niger	Maradi	1
Niger	Tillaberi	4
Benin	Parakou	2
Burkina Faso	Ouagadougou	1
Mali	Bamako	7
Mauritania	Nouakchott	1
Togo	Adeta	1
Total		109

## 3. Results

### 3.1. Virus Isolation and Sequencing

A total of seven NDV isolates were sequenced from the samples collected within the Northeastern region of Nigeria. Full‐length F and HN gene sequences were successfully obtained for all the isolates, and analysis of the F gene cleavage site revealed the presence of the typical virulent motif ^112^RRRKRF^117^, characteristic of velogenic NDV strains, which are associated with high pathogenicity and responsible for most of the recent ND outbreaks in Nigeria.

### 3.2. Phylogenetic Analyses

**Figure 1:**
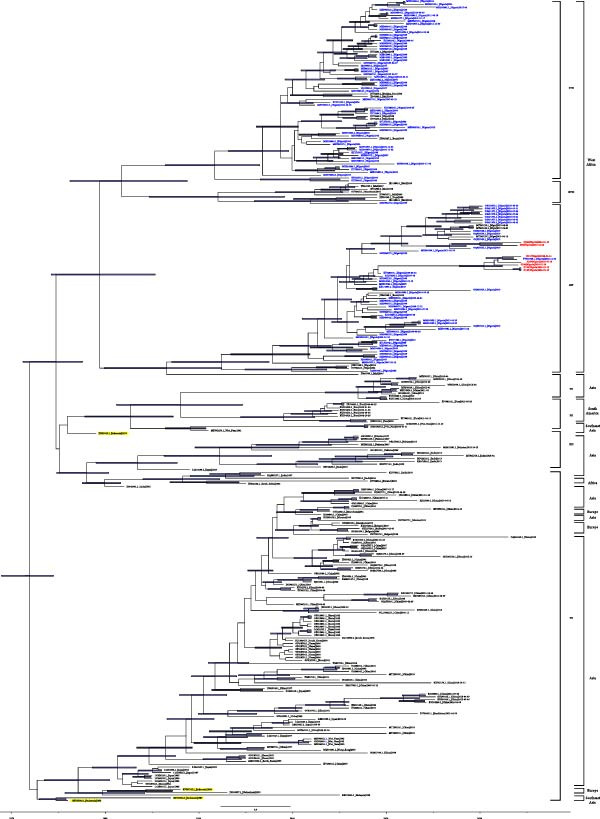
The maximum clade credibility tree, constructed from the sequences of the seven‐field samples and 279‐retrieved NDV full‐length F genes. The viruses isolated in this study were presented in red font. Retrieved Nigerian isolates were shown in blue font. Isolates from Indonesia were highlighted in yellow. Blue node bars represent median height range of 95% HPD.

The ML analysis of the full‐length F gene showed 80%–100% nucleotide identity among NDV strains from Nigeria (Figures S2–S4), and all seven isolates belong to subgenotype XIV.2 of class II [14]. The ML tree of 428 HN gene sequences revealed that West African NDV isolates formed a distinct cluster of class II genotypes XIV and XVII, while showing evolutionary relationships with several Asian NDV isolates, most notably strains from Indonesia (Figure S5). The MCC phylogenetic analysis of the F gene suggests that class II NDV genotypes XIV, XVII, and XVIII were likely introduced into Nigeria from Southeast Asia. These genotypes have since become established in Nigeria, evolving locally and contributing to ongoing transmission, including spread to neighboring countries. Based on the available sequence data, it seems genotype XVII (KUDU 113 strain) has not been detected in Nigeria since 2015 (Figure 1).

The evolutionary rates estimated for all the F genes in this study are 1.33 × 10^−3^ substitutions/site/year (95% highest posterior density [HPD]: 1.19 × 10^−3^–1.47 × 10^−3^). The estimated time to the most recent common ancestor (tMRCA) was 24 May, 1979 for clusters of class II genotypes XIV, XVII, and XVIII, 28 September, 1981 for genotypes XVII and XVIII, and 13 July, 1986 for genotype XIV (Table 3). This indicates that class II NDV strains of genotypes XVII and XVIII probably emerged before genotype XIV (Table 3).

**Table 3:** Estimated tMRCA for the F gene sequences from this study with 95% HPD.

Class II genotype cluster	tMRCA ^∗^	95% HPD interval		Posterior probability
		Begin	End	
Root age	20 Sept., 1974	13 March, 1971	18 July, 1977	1
XIV, XVII, XVIII	24 May, 1979	19 March, 1974	26 April, 1985	0.99
XVII, XVIII	28 Sept., 1981	20 March, 1976	29 Sept., 1988	0.68
XIV	13 July, 1986	15 Nov., 1979	6 June, 1992	1

Abbreviation: HPD, highest posterior density.

^∗^The tMRCA was calculated using BEAST tree with most recent tip date 2024.3784.

### 3.3. Selection Pressure Analysis

**Figure 2:**
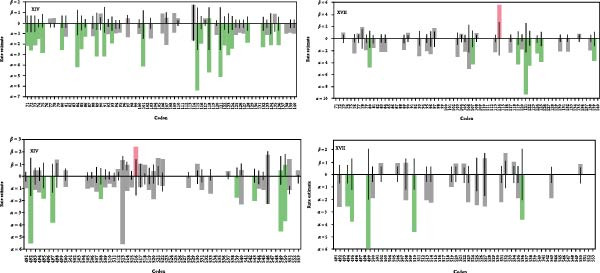
Maximum likelihood estimates of synonymous (*α*) and non‐synonymous rates (*β*) at each site shown as bars on the fixed effects likelihood (FEL) graph of full‐length F gene NDV genotype XIV and XVII. The horizontal line represents the estimates under the null model (*α* = *β*). Red Bar is diversifying (516 in XIV and 114 in XVII), green bar is purifying, grey bar is neutral, and without bar is invariable.

The aligned full‐length F gene sequences from genotypes XIV and XVII were separately analyzed to evaluate the selection pressure on each of the two NDV genotypes. For genotype XIV, the FEL method identified 120 sites under purifying selection (*p* ≤ 0.1), with codon 516 exhibiting diversifying selection (Figure 2). Key codons under significant negative pressure (*p* ≤ 0.1) included 62, 115, 150, 318, 361, and 492. The FUBAR analysis confirmed widespread purifying selection (249 codon sites), with 1 positive selection site in 516 (Figures S6–S8). The MEME detected episodic positive selection at codons 301, 516, and 518 (Figure S9), while SLAC estimated a mean dN/dS (*ω*) ratio of 0.157, with 54 negative selection sites out of 553 codons (Figure S10). For genotype XVII, FEL identified 53 purifying selection sites (*p* ≤ 0.1), with codon 114 under diversifying selection (Figure 2). Key negatively selected codons (*p* ≤ 0.1) included 3, 121, 163, 243, 305, 474, and 499. The FUBAR analysis yielded a notable pervasive negative/purifying selection in 78 codon sites with one site (114) under positive selection (Figure S11). The MEME method also detected episodic positive selection at codon 114 with a *p*‐value of 0.05 (Figure S12), while the SLAC analysis reported a mean dN/dS (*ω*) ratio of 0.140, with 17 negative selection sites out of the 553 codon sites in the alignment (Figure S13). Generally, the F gene in Nigerian NDV isolates of genotypes XIV and XVII predominantly undergoes negative selection, with only codons 516 in genotype XIV and 114 in genotype XVII, which are consistently under positive selection across the four methods.

### 3.4. Nextstrain Phylogeographic Analysis

**Figure 3:**
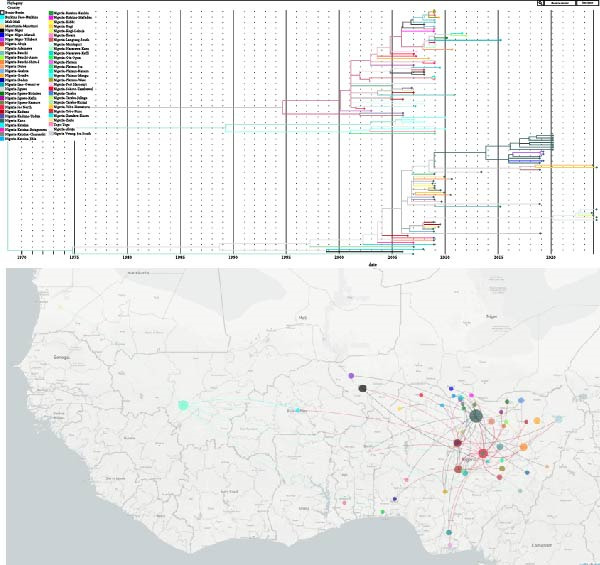
Phylogenetic tree and geographic spread map of West African NDV isolates, generated using Nextstrain.

The Nextstrain tree features many color‐coded tips representing different Nigerian states or West African countries and deep splits between major clades with some long branch lengths (Figure 3). It indicates both undetected long‐term evolution and recent detection. The geographic transmission map points to Central Nigeria as a key hub for virus spread. The analysis generally highlights genetic diversity with multiple co‐circulating lineages and local adaptation events. Some strains seem to have persisted over several years, suggesting ongoing local evolution. The data also indicate reintroductions from neighboring regions, underscoring an active viral evolution and widespread co‐circulation across West Africa (Figure 3).

## 4. Discussion

In the past decades, poultry farmers in Nigeria have faced challenges, with insurgency, climate change, and endemic diseases like ND severely impacting their businesses and livelihoods [3, 19]. The first ND outbreak in Nigeria was reported around 1953 by Hill et al. [38], and the disease has continued to re‐emerge with time, accompanied by the appearance of distinct regional strains such as genotypes XIV, XVII, and XVIII [16, 39]. The genotype XVII (isolate KU058680) was among the first alarming strain identified in Nigeria around 1992 and became a threat to poultry health due to its virulence [40]. As a result, Nigeria emerged as a hotspot for NDV circulation, potentially contributing to the spread of the virus to neighboring countries and even as far as the United States [16, 18]. However, the precise route and origin of NDV introduction into Nigeria remain unclear [39, 41]. Based on the time‐scaled Bayesian phylogenetic analysis of F gene sequences in this study, the recently circulating NDV genotypes XIV, XVII, and XVIII in Nigeria appear to have probably originated from Indonesia, one of the regions where NDV was first reported [42]. The MCC tree analysis (Figure 1) reveals that Nigerian NDV isolates share an evolutionary link with the extinct genotype XIII (JX393313.1). Despite presumed extinction, a novel genotype XIII was later discovered in India [43], and some of its strains possess a similar cleavage site motif (RRRKRF) to that of the subgenotype XIV.2 isolates identified in this study (Table 1). The strain (JX393313.1) that is genetically close to the suspected ancestor was isolated from a mosquito in Indonesia in 1977 during an arbovirus surveillance program [44]. Although ND is not a vector‐borne disease, isolating the virus from a mosquito raises many questions [44]. The mosquito pool was processed in a laboratory also handling avian influenza samples, and the isolate was lyophilized with other NDV samples, raising the possibility of cross‐contamination [44].

Indonesia, renowned for its rich wild bird diversity, poses a potential risk for disease transmission due to the widespread trade and movement of these birds [45]. From the BEAST tree (Figure 1), the early‐branching NDV isolates (KF767106.1|1976‐Parrot, KF767104.1|1987‐Cockatoo, KF767105.1|1988‐Lory) demonstrate a close epidemiological connection to *Psittacidae* family in Indonesia, and this avian group may represent a potential origin of the newly emerged NDV strains in Nigeria. Moreover, poultry trade from Southeast Asia to West Africa may have led to the spread of such viruses due to weaker biosecurity enforcement in West Africa [45, 46], and the added risk could be from Palearctic migratory birds mixing with free‐range village chickens in northern Nigeria [47]. In addition, the first isolation of NDV from a parrot in Nigeria in January, 1979 [48], aligns closely with the estimated tMRCA of the newly emerged class II NDV genotypes (XIV, XVII, and XVIII), dated 24 May, 1979 (Table 3). Many studies in Nigeria have also confirmed the presence of NDV in captive and free‐flying wild birds [49–55]. Also, among the clustered class II NDV subgenotype XIV.2 in this study, MH996923.1 was isolated from a migratory raptor in Taraba State (Figure 1). Furthermore, feral migratory birds were thought to have spread another NDV strain (GU585905), which shared 100% similarity with the Indonesian isolate (JX393313.1) in question [44, 56]. Exotic birds may be more resistant to NDV than chickens and may serve as silent carriers, probably aiding the introduction of novel NDV from Southeast Asia into Nigeria [2, 50, 57]. This evolutionary connection suggests a possible ancestral lineage between the now‐prevalent Nigerian strains and earlier Southeast Asian isolates, reinforcing the transcontinental transmission dynamics of NDV.

The molecular analysis from this study identified the seven samples as class II NDV subgenotype XIV.2 (Figure 1). This finding aligns with recent studies conducted in Nigeria, where almost all sequenced samples were classified as class II NDV genotype XIV.2, indicating continued circulation of this particular strain in the region [19, 20, 58, 59]. It seems class II NDV genotype XVII disappeared after 2015, probably due to massive vaccination with thermostable vaccine strains (I‐2 or V4 https://kyeemafoundation.org/history-of-the-i-2-nd-vaccine/) in the early 2000s [53]. Studies have shown that these improved LaSota‐like vaccines have provided protection to the class II NDV genotype XVII [60–62]. This could be as a result of class II NDV genotype XVII having similar antigenic cleavage sites (RQKRFIGA) with LaSota (RQGRLIGA) and Komorov (RQKRFIGA) while class II NDV genotype XIV.2 remains entirely different (RRKRFVGA) [63].

The Nextstrain analysis of NDV isolates from this study, alongside regionally related strains in West Africa, reveals lineage diversity with distinct genotypes co‐circulating (Figure 3). Deep branching and long divergence times in the phylogenetic tree reflect prolonged viral evolution and numerous introductions spanning decades [15]. This evolutionary history underpins regional sequence clustering, indicating local adaptation with frequent viral movement between states or nearby countries, as shown by the transmission map (Figure 3). Geographically, Central Nigeria, including states such as Kano and Plateau, appears to serve as major transmission hubs, radiating virus to other regions. This pattern likely reflects the concentration of poultry production and LBMs in these areas [19, 53, 58]. The detection of ND virus in both wild and captive birds in Central Nigeria highlights a potential reservoir, further indicating the sustained prevalence of the disease in the region [50]. The presence of the NVRI in the region has also contributed to increased NDV research activity [18, 19, 58, 64–66]. Despite frequent NDV isolation in the region, this analysis is constrained by scarce Nigerian and West African sequences, with most available data limited to partial F gene sequences.

These novel NDV genotypes in Nigeria may have probably derived from class II genotype VII or XIII due to point mutations since all are previously classified as either lineage five or seven [14]. For example, isolate‐GU585905 that clustered with genotype XIII is previously classified as VIIb by Linde et al. [56], and isolate‐JF966385.1 is mistaken as genotype XIV.1 in the GenBank submission while it clusters under genotype XVII.1. Over and beyond, the NDV genotype XIV.2 circulating in Nigeria exhibits a high intracerebral pathogenicity index (ICPI) of up to 1.84 [18, 60] and this reflects codon‐specific diversification of virulence of such NDV strains [67] despite predominantly conserving viral proteins to maintain viral fitness within the host (Figure 2). Notably, most F gene codon sites in NDV genotypes XIV and XVII from Nigeria are under negative selection, indicating conserved viral functions. This suggests both genotypes are under stabilizing evolutionary pressure, potentially reflecting regionally host adaptation. However, the positive selection at codon 114 in NDV genotype XVII F protein (Figure 2) suggests evolutionary flexibility, and mutation at the site alters its virulence [68, 69]. In contrast, codon 115 undergoes strong negative selection in genotype XIV F gene (Figure 2), highlighting its essential role in maintaining cleavage efficiency and probably high pathogenicity [68, 69]. These findings emphasize the dynamic nature of NDV evolution especially in Nigeria and the need for continued surveillance to monitor strain variation, assess potential cross‐border transmission, and guide effective disease control measures.

## 5. Conclusion

This study characterized seven NDV isolates, all belonging to class II subgenotype XIV.2. Based on the Bayesian phylogenetic inference, the recently circulating class II NDV genotypes XIV, XVII, and XVIII in Nigeria likely introduced from Southeast Asia longtime ago, possibly introduced through wild bird migration or trading. This is supported by the first recorded NDV case in a parrot in Nigeria in 1979, which aligns with the estimated tMRCA for these emerging genotypes. Genotype XVII appears to have disappeared in Nigeria after 2015, likely due to widespread vaccination with thermostable vaccine strains in the early 2000s. Selection pressure analysis revealed strong purifying selection, indicating conserved viral functions and ongoing host adaptation. These findings highlight the need for continuous molecular surveillance and tailored vaccination strategies. A practical and effective solution would be the deployment of thermostable vaccines that matched the local genotypes, combined with improved biosecurity and monitoring of wild bird reservoirs to prevent future introductions.

## Ethics Statement

The ethical clearance to conduct the research was obtained from the Research Ethics Committee of Sokoine University of Agriculture, Tanzania, with reference number SUA/ADM/R.1/8/1065 and the Ethics Committee of Faculty of Veterinary Medicine, University of Maiduguri under the reference number FVM/UNIMAID/AUEC/2023/006. All sample collection procedures were carried out in accordance with the Ethics Committee’s guidelines.

## Conflicts of Interest

The authors declare no conflicts of interest.

## Author Contributions

**Mohammed Usman Sajo**: conceptualization, methodology, software, validation, formal analysis, data curation, writing – original draft, writing – review and editing, visualization, and funding acquisition. **Dongyeop Lee**: methodology, software, and visualization. **Jean Nepomuscene Hakizimana**: conceptualization, writing – review and editing, and project administration. **Augustino Chengula**: conceptualization, writing – review and editing, supervision, and project administration. **Abdul-Dahiru El-Yuguda**: conceptualization, writing – review and editing, and supervision. **Dong-Hun Lee** and **Gerald Misinzo**: conceptualization, writing – review and editing, supervision, resources, project administration, and funding acquisition.

## Funding

This study was funded by the Partnership in Applied Sciences, Engineering and Technology (PASET) under the Regional Scholarship and Innovation Fund (RSIF) Grant to SACIDS Africa Centre of Excellence for Infectious Diseases of Humans and Animals in Southern and East Africa (SACIDS‐ACE) at Sokoine University of Agriculture (SUA) with Project Grant Number P165581. Mohammed Usman Sajo is a recipient of the PASET‐RSIF Doctoral Scholarship at SUA and an additional DOCTAS grant (Grant Number G‐22‐59858) through the collaborative partnership between the International Centre of Insect Physiology and Ecology (icipe) and the Carnegie Corporation of New York.

## Supporting Information

Additional supporting information can be found online in the Supporting Information section.

## Supporting information


**Supporting Information 1** Table S1: The metadata of the 279 retrieved GenBank full‐length sequences used in this study. Table S2: The details of the NDV full‐length F gene sequences of the West African isolates used for the Nextstrain analysis.

**Supporting Information 2** Figure S1: Root to tip of the maximum likelihood (ML) tree of the 286 full‐length F gene sequences from this study. Figure S2: The ML tree of 286 full‐length F gene sequences. The seven study samples are marked in red. Figure S3: The ML tree of genotype XIV from Nigeria. The seven study samples are marked in red. Figure S4: The ML tree of genotype XVII from Nigeria. Figure S5: The ML tree of 428 full‐length HN gene sequences. Figure S6: Genotype XIV codon site 115 negative/purifying selection by fast, unconstrained Bayesian AppRoximation (FUBAR) graph showing the posterior distribution over the discretized rate grid. Figure S7: Genotype XIV codon site 516 positive/diversifying selection by FUBAR graph showing the posterior distribution over the discretized rate grid. Figure S8: Genotype XIV codon site 517 neutral by FUBAR graph showing the posterior distribution over the discretized rate grid. Figure S9: Genotype XIV mixed effects model of evolution (MEME) site plot showing episodic diversifying selection. Figure S10: Genotype XIV selection pressure single‐likelihood ancestor counting (SLAC) site graph showing a predominant negative selection. Figure S11: Genotype XVII codon site 114 positive/diversifying selection by FUBAR graph. Figure S12: Genotype XVII MEME site plot showing episodic diversifying selection. Figure S13: Genotype XVII selection pressure SLAC site graph showing a predominant negative selection.

## Data Availability

The raw NGS reads are deposited in the NCBI database under the BioProject Accession number, PRJNA1321608. The assembled complete F gene sequence data have been deposited in the NCBI database under the GenBank Accession numbers, PX095534‐PX095540. The assembled complete HN gene sequences were also deposited to the GenBank under the Accession numbers, PX376745‐PX376751.
