# Supplementary material for: Evolution and Spread of Regionally Adapted Newcastle Disease Virus Isolates From Live Bird Markets in Nigeria, 2023–2024
Source: Transbound Emerg Dis. 2026 Jan 24;2026:8829822. doi: 10.1155/tbed/8829822 (PMC12831130; doi:10.1155/tbed/8829822)
Supplement: Supplementary file 1 — Supporting Information 1 Table S1: The metadata of the 279 retrieved GenBank full‐length sequences used in this study. Table S2: The details of the NDV full‐length F gene sequences of the West African isolates used for the Nextstrain analysis. [file TBED-2026-8829822-s002.docx]

**SUPPLEMENTARY FILE 1**

**Evolution and Spread of Regionally Adapted Newcastle Disease Virus Isolates from Live Bird Markets in Nigeria, 2023-2024**

Mohammed Usman Sajo^1,2,3,4*^, Dongyeop Lee^5^, Jean Nepomuscene Hakizimana^2^, Augustino Chengula^1^, Abdul-Dahiru El-Yuguda^3^, Dong-Hun Lee^4,5*^, Gerald Misinzo^1,2*^

^1^Department of Microbiology, Parasitology and Biotechnology, College of Veterinary Medicine and Biomedical Sciences, Sokoine University of Agriculture, Morogoro, 67152, Tanzania

^2^OR Tambo Africa Research Chair for Viral Epidemics, SACIDS Foundation for One Health, Sokoine University of Agriculture, Morogoro, 67152, Tanzania

^3^Animal Virus Research Laboratory, Department of Veterinary Microbiology, Faculty of Veterinary Medicine, University of Maiduguri, Maiduguri, 600001, Nigeria

^4^Konkuk University Zoonotic Disease Research Center, College of Veterinary Medicine, Konkuk University, Seoul, 05029, Republic of Korea

^5^Wildlife Health Laboratory, College of Veterinary Medicine, Konkuk University, Seoul, 05029, Republic of Korea

*Corresponding authors; MUS: [sajom@unimaid.edu.ng](mailto:sajom@unimaid.edu.ng), DHL: [donghunlee@konkuk.ac.kr](mailto:donghunlee@konkuk.ac.kr), GM: [gerald.misinzo@sacids.org](mailto:gerald.misinzo@sacids.org)

The responsible corresponding author: Gerald Misinzo, Department of Microbiology, Parasitology and Biotechnology, College of Veterinary Medicine and Biomedical Sciences, Sokoine University of Agriculture, Morogoro, 67152, Tanzania

Suppl. Table 1: The metadata of the 279 retrieved GenBank full-length F gene sequences used in this study

| Accession number | Collection date | Geo_loc_name | Host | Isolate name | Isolation source |
| --- | --- | --- | --- | --- | --- |
| EU140947 | 2000 | South Korea |  | SNU-0202 |  |
| EU140949 | 2005 | South Korea |  | SNU-5070 |  |
| FJ480788 | 2006 | China | wild bird | NDV/WB/CH/HLJ001/06 |  |
| FJ480790 | 2006 | China | chicken | CK/CH/HLJ/1/06 |  |
| FJ480796 | 2001 | China | chicken | CK/CH/HN/1/01 |  |
| FJ480798 | 2004 | China | chicken | CK/CH/HN/1/04 |  |
| FJ480801 | 2005 | China | chicken | CK/CH/JL/1/05 |  |
| FJ480802 | 2006 | China | chicken | CK/CH/JL/1/06 |  |
| FJ480804 | 2006 | China | chicken | CK/CH/JL/2/06 |  |
| FJ480806 | 2003 | China | chicken | CK/CH/JL/4/03 |  |
| FJ480813 | 2005 | China | chicken | CK/CH/NX/1/05 |  |
| FJ480818 | 2006 | China | chicken | CK/CH/NX/5/06 |  |
| FJ480822 | 2006 | China | goose | Go/CH/HLJ/1/06 |  |
| FJ480824 | 2005 | China | mallard duck | Md/CH/GD/1/05 |  |
| FJ872531 | 2002 | China: Fujian | Muscovy duck | Muscovy duck/China(Fujian)/FP1/02 |  |
| GQ245781 | 2008 | China | chicken | CZ-8-06-Ch |  |
| GQ245783 | 2007 | China | chicken | HA-2-07-Ch |  |
| GQ245787 | 2007 | China | chicken | HA-14-07-Ch |  |
| GQ255639 | 26-Feb-09 | China | goose | GPMV-SH |  |
| FJ772446 | 2006 | Nigeria | avian | avian-913-1-Nigeria-2006 |  |
| FJ772449 | 2006 | Nigeria | avian | avian-913-33-Nigeria-2006 |  |
| FJ772452 | 2006 | Niger | chicken | chicken-1377-8-Niger-2006 |  |
| FJ772455 | 2006 | Mauritania | avian | avian-1532-14-Mauritania-2006 |  |
| FJ772458 | 2008 | Burkina Faso | chicken | chicken-2415-361-Burkina Faso-2008 |  |
| FJ772469 | 2008 | Niger | chicken | chicken-2602-348-Niger-2008 |  |
| FJ772472 | 2008 | Niger | chicken | chicken-2602-468-Niger-2008 |  |
| FJ772481 | 2008 | Niger | chicken | chicken-2602-625-Niger-2008 |  |
| FJ772486 | 2008 | Nigeria | avian | avian-3724-6-Nigeria-2008 |  |
| FJ772494 | 2008 | Burundi | chicken | chicken-4132-20-Burundi-2008 |  |
| GU227738 | 7-Feb-07 | Serbia | dove | NDV/Serbia/749/2007 |  |
| GU182323 | 2008 | Pakistan | chicken | ND43 |  |
| GU182331 | 2007 | Pakistan | chicken | ND33 |  |
| GU332646 | 2002 | Viet Nam | duck | APMV-1/duck/Vietnam/Long Bien/78/2002 |  |
| HM748944 | 17-Jan-07 | China | broiler chicken | chicken/China/SD2/2007 |  |
| HQ589257 | 1997 | India | chicken | Bareilly |  |
| JF343538 | 2003 | China |  | ND/03/018 |  |
| HQ917080 | 2008 | China | goose | A/goose/Guangxi/1/2008 |  |
| JF340367 | Oct-02 | China | goose | JSG0210 |  |
| JF966385 | 2008 | Mali | chicken | 2008_Mali_ML007_08 | cloacal swab |
| JF966386 | 2007 | Mali | chicken | 2007_Mali_ML029_07 | cloacal swab |
| JF966387 | 2009 | Mali | chicken | 2009_Mali_ML008 | cloacal swab |
| JF966388 | 2008 | Mali | chicken | 2008_Mali_ML225_08 | tracheal swab |
| JF966389 | 2007 | Mali | Guinea fowl | 2007_Mali_ML038_07 | tracheal swab |
| JN400896 | 16-Jan-11 | China | chicken | Chicken/China/SDSG01/2011 |  |
| JN599167 | 1999 | China | penguin | BP01 |  |
| JN986837 | 1993 | Netherlands | chicken | 152608 |  |
| JN872165 | 2006 | USA | chicken | Chicken/Niger/VIR 1377-7/2006 |  |
| JN942034 | 1995 | South Africa | ostrich | Ostrich/South Africa/45445-3/1995 |  |
| JN942041 | 1982 | India | cockatoo | Cockatoo/India/7847/1982 |  |
| JQ039386 | 2008 | Nigeria | Gallus gallus | chicken/Nigeria/VRD08-36/2008 |  |
| JQ039390 | 2007 | Nigeria | Gallus gallus | chicken/Nigeria/VRD07-233/2007 |  |
| JQ039393 | 2007 | Nigeria | Gallus gallus | chicken/Nigeria/VRD07-141/2007 |  |
| JQ039394 | 2007 | Nigeria | Gallus gallus | chicken/Nigeria/VRD07-410/2007 |  |
| JN618348 | 1997 | China | chicken | XJ-2/97 |  |
| JN618349 | 2005 | China | chicken | JS-3-05-Ch |  |
| JX390609 | 2009 | Togo: Akodessewa | chicken | NDV/chicken/Togo/AKO18/2009 | oral swab |
| FJ754272 | Apr-00 | China | duck | WF00D | liver |
| FJ754273 | Mar-00 | China | goose | WF00G | liver |
| KC152048 | 2011 | China | goose | GD450/2011 |  |
| KC152049 | 2010 | China | goose | GD1003/2010 |  |
| JX518885 | 2010 | Mali | chicken | 2010_Mali_ML57051T |  |
| JX518886 | 2010 | Mali | chicken | 2010_Mali_ML57072T |  |
| JX546245 | 2009 | Benin | chicken | NDV/chicken/Benin/463MT/2009 |  |
| JX546247 | 2009 | Benin | chicken | NDV/chicken/Benin/488MT/2009 |  |
| KC551967 | 3-Feb-10 | China | goose | goose/Guangdong/2010 |  |
| KC542895 | Nov-06 | China | Gallus gallus | Chicken/China/Hebei/01/2006 |  |
| JX393313 | 1979 | Indonesia | Culex tritaeniorhynchus pool | JKT1997 | pool of mosquitoes |
| KC750155 | 2009 | China | duck | Duck/CH/GD/YF-09 |  |
| KC568204 | Jul-09 | Nigeria | Columba livia (Columba livia (pigeon)) | pigeon/Nigeria/ZM/KN/PG01/N1/688/2009 |  |
| KC568205 | Aug-09 | Nigeria | Numida meleagris (G-fowl) | NG-705/KD.TW.7C |  |
| KC568206 | Aug-09 | Nigeria | Numida meleagris (G-fowl) | NG-706/JG.KZ.14T |  |
| KC568208 | Jun-09 | Nigeria | Gallus gallus (chicken) | NG-710/GM.PLBM.10-12T |  |
| AB853929 | Jun-02 | Japan:Miyagi | Gallus gallus domesticus | APMV1/chicken/Japan/Miyagi/AGT/2002 |  |
| KF208469 | 2013 | China | chicken | Ch/SD883/13 |  |
| AB853927 | Sep-99 | Japan:Ibaraki | Gallus gallus domesticus | APMV1/chicken/Japan/Ibaraki/SG106/1999 |  |
| KF727980 | 2006 | India | chicken | Bareilly |  |
| KF740478 | 2003 | India: TN | Japanese quail | NDV2K35/CH/TN/2003 |  |
| KF442614 | 2006 | Nigeria | chicken | Nigeria/228-7/2006 |  |
| KF767104 | 1987 | Indonesia | cockatoo | Cockatoo/Indonesia/1988/87-36724-524 |  |
| KF767105 | 1988 | Indonesia | lory | Lory/indonesia/1988/88-08989-523 |  |
| KF767106 | 1976 | Indonesia | parrot | Parrot/Indonesia/1976/C300(19625)-520 |  |
| KC853019 | 25-Feb-06 | China | crested ibis | NDV/crested ibis/China/Shaanxi06/2006 |  |
| KJ577585 | 2010 | India | chicken | NDV/Chicken/Bareilly/01/10 | lung homogenate |
| KJ528559 | 2-Mar-14 | China | goose | NA-1M |  |
| KJ607169 | 2006 | China | goose | go/CH/LHLJ/1/06 |  |
| KJ782375 | 1997 | China | goose | go/CH/GD-QY/1997 |  |
| KJ525676 | Jun-06 | China | goose | Go/GD/GZ/2006 |  |
| KJ525679 | Sep-08 | China | duck | Du/SD/DZ/2008 |  |
| KJ525690 | 14-Apr-13 | China | chicken | Ch/SD/YT/2013 |  |
| KJ525717 | 8-Jun-08 | China | chicken | Ch/SD/MH/2008 |  |
| KM977903 | 12-May-05 | China | duck | HB/1/05/Dk |  |
| KM885167 | 2005 | China | duck | Md/CH/LGD/1/2005 |  |
| KT948996 | 1-Jun-09 | Nigeria: Kogi | Anas platyrhynchos | duck/Nigeria/NG-695/KG.LOM.11-16/2009 |  |
| KR074406 | 2005 | Malaysia | Gallus gallus | MB076/05 | allantoic fluid |
| KT889365 | Oct-14 | China | mink | mNDV-01-HLJ |  |
| KU594613 | 22-Nov-04 | Peru | chicken | chicken/Lima-Peru/40931/2004 |  |
| KU594614 | 30-Sep-04 | Peru | chicken | chicken/Lima-Peru/40785/2004 |  |
| KU594615 | 4-Jan-05 | Peru | chicken | chicken/Apurimac-Peru/50009/2005 |  |
| KU594616 | 26-Oct-04 | Peru | chicken | chicken/Lurin-Peru/40871/2004 |  |
| KU140419 | 2006 | China | Gallus gallus | ck/CH/LHLJ/1/06 |  |
| KU200245 | 3-Apr-15 | China | quail | Quail/China/Jilin/JY02/2015 |  |
| KU200246 | 6-Mar-15 | China | chicken | Chicken/China/Jilin/YJ05/2015 |  |
| KU200248 | 6-Apr-14 | China | pigeon | Pigeon/China/Heilongjiang/LS03/2014 |  |
| KU295450 | 2006 | Bulgaria | chicken | chicken/Bulgaria/Juper/2006 |  |
| KU295452 | 2007 | Bulgaria | chicken | chicken/Bulgaria/Vidno/2007 |  |
| KU710274 | 2008 | Bulgaria | chicken | chicken/Bulgaria/Kazatsite/2008 |  |
| KU710277 | 2011 | Ukraine | pigeon | pigeon/Ukraine/Simferopol/2-26-11/2011 |  |
| KU295454 | 2003 | Ukraine | chicken | chicken/Ukraine/Lyubotyn/961/2003 |  |
| KX061544 | 2013 | India | Eos bornea (Red Lori) | Redlori/CHN/2013 |  |
| KY171989 | 2010 | Nigeria | chicken | chicken/Nigeria/VRD10/143/N68/913/2010 |  |
| KY171991 | 2004 | Nigeria | quail | quail/Nigeria/VRD17/04/N2/861/2004 |  |
| KY171992 | 2009 | Nigeria | chicken | chicken/Nigeria/JN/469/N44/892/2009 |  |
| KY171993 | 2009 | Nigeria | chicken | chicken/Nigeria/VRD09/031/N23/715/2009 |  |
| KY171994 | 2009 | Nigeria | chicken | chicken/Nigeria/VRD09/001/N19/714/2009 |  |
| KY171995 | 2006 | Nigeria | chicken | chicken/Nigeria/VRD124/06/N11/867/2006 |  |
| KX372710 | 2009 | India | chicken | NDV/Chicken/Nagpur/07/09 | lung |
| KX765177 | 24-Apr-11 | China | duck | DU-FJCL117 |  |
| KX765178 | 7-Aug-12 | China | duck | DU-JXNC35 |  |
| KX765179 | 11-Oct-14 | China | duck | DU-FJ-241 |  |
| MF278926 | May-11 | China | goose | GS/FS/GM/4/2011 |  |
| MF278927 | Apr-13 | China | goose | GS/FS/SS/292/2013 |  |
| MF278928 | May-11 | China | goose | GS/ZQ/JL/12/2011 |  |
| MF278929 | Mar-11 | China | goose | GS/ZQ/XG/17/2011 |  |
| MF278930 | Oct-14 | China | goose | GS/ZQ/XG/502/2014 |  |
| MF278933 | Apr-16 | China | muscovy duck | MDK/MM/GZ/884/2016 |  |
| MH019281 | 13-Oct-15 | Pakistan |  | PK1 |  |
| MG867723 | 2008 | China | broiler chicken | G7 |  |
| MF422126 | 2013 | India: Hyderabad | chicken | 478-13A |  |
| MF422128 | 2013 | India: Udumalepet | chicken | 248-13A2 |  |
| MG869270 | 11-Jan-08 | Viet Nam: Bac Giang | chicken | NDVQG |  |
| MG869271 | 10-Nov-11 | Viet Nam: Khanh Hoa | chicken | NCXKH |  |
| MH392222 | 2007 | Pakistan | chicken | chicken/Pakistan/SPVC/Karachi/27/558/2007 |  |
| MH392223 | 2007 | Pakistan | chicken | chicken/Pakistan/SPVC/Karachi/33/556-XIII/2007 |  |
| MH392225 | 2009 | Nigeria | chicken | chicken/Nigeria/KD/TW/03T/N45/720/2009 |  |
| NC_039223 | Dec-08 | China | laying duck | JSD0812 |  |
| MH092804 | 2003 | Nigeria | chicken | chicken/Nigeria/VRD67/03 (N1)/2003 |  |
| MH092805 | 2006 | Nigeria | chicken | chicken/Nigeria/VRD41/06 (N3)/2006 |  |
| MH092806 | 2006 | Nigeria | chicken | chicken/Nigeria/VRD144/06 (N6)/2006 |  |
| MH092807 | 2007 | Nigeria | chicken | chicken/Nigeria/VRD07/338 (N18)/2007 |  |
| MH092813 | 2006 | Nigeria | chicken | chicken/Nigeria/VRD298/06 (N7)/2006 |  |
| MH092814 | 2006 | Nigeria | chicken | chicken/Nigeria/VRD309/06 (N8)/2006 |  |
| MH092815 | 2007 | Nigeria | chicken | chicken/Nigeria/VRD07/290 (N16)/2007 |  |
| MH092816 | 2009 | Nigeria | local chicken | local chicken/WaseNigeria/WAS/447 (N39)/2009 |  |
| MH092817 | 2009 | Nigeria | local chicken | local chicken/WaseNigeria/WAS/465 (N40)/2009 |  |
| MH092821 | 2009 | Nigeria | local chicken | local chicken/Jos NorthNigeria/JN/457 (N57)/2009 |  |
| MH092822 | 2008 | Nigeria | chicken | chicken/Nigeria/VRD08/98 (N63)/2008 |  |
| MH092823 | 2008 | Nigeria | chicken | chicken/Nigeria/VRD08/201 (N64)/2008 |  |
| MH092824 | 2012 | Nigeria | chicken | chicken/Nigeria/VRD12/013 (N70)/2012 |  |
| MH105250 | Oct-15 | China | duck | duck/Shandong/142/2015 |  |
| MH392227 | 2009 | Nigeria | chicken | chicken/Nigeria/OOT/4/1/N69/914/2009 |  |
| MH092808 | 2009 | Nigeria | turkey | turkey/Nigeria/JN/327(N24)/2009 |  |
| MH092809 | 2009 | Nigeria | duck | duck/Langtang South/Nigeria/LTS/08(N25)/2009 |  |
| MH092810 | 2009 | Nigeria | duck | duck/Kanam/Nigeria/KN/399(N26)/2009 |  |
| JN800306 | 2008 | Peru | poultry | chicken/Peru/1918-03/603/2008 |  |
| MK006001 | 2003 | Hong Kong | chicken | chicken/Hong-Kong/SV-03-1933.5/376/2003 |  |
| MK006002 | 1995 | South Korea | chicken | chicken/South Korea/95-119/513/1995 |  |
| MK006022 | 2002 | Viet Nam | chicken | chicken/Vietnam/482/2002 |  |
| MK006024 | 2002 | Viet Nam | pigeon | pigeon/Vietnam/484/2002 |  |
| MH996905 | 2008 | Nigeria | Gallus gallus | local chicken/Nigeria/VRD08/81/N31/933/2008 |  |
| MH996906 | 2008 | Nigeria | chicken | chicken/Nigeria/VRD08/296A/N32/934/2008 |  |
| MH996907 | 2009 | Nigeria | Gallus gallus | local chicken/Nigeria/JN/458/N36/935/2009 |  |
| MH996908 | 2009 | Nigeria | chicken | chicken/Nigeria/VRD09/068/N92/942/2009 |  |
| MH996909 | 2012 | Nigeria | chicken | chicken/Nigeria/VRD12/210/N100/947/2012 |  |
| MH996980 | 30-May-13 | Nigeria | chicken | chicken/Nigeria/Vwang/VRD97/67/2013 |  |
| MH996981 | Apr-15 | Nigeria | chicken | chicken/Nigeria/VRD798-XIV/15/798/2015 |  |
| MH996982 | Apr-15 | Nigeria | chicken | chicken/Nigeria/Owerri-west/VRD798- XVII/15/798/2015 |  |
| MH996984 | 18-Aug-11 | Nigeria | chicken | chicken/Nigeria/Katsina/KT-MG-C2-3/75/2011 |  |
| MH996986 | 21-Dec-09 | Nigeria | chicken | chicken/Nigeria/VRD599/59/2009 |  |
| MH996987 | 21-Dec-09 | Nigeria | chicken | chicken/Nigeria/Kogi/VRD578/60/2009 |  |
| MH996988 | 4-Nov-10 | Nigeria | chicken | chicken/Nigeria/Keffu/NS-KF-C13-17/90/2010 |  |
| MH996989 | 28-Aug-10 | Nigeria | chicken | chicken/Nigeria/Dutse/JG-DT-C32-36-XIV/102/2010 |  |
| MH996990 | 28-Aug-10 | Nigeria | chicken | chicken/Nigeria/Dutse/JG-DT-C32-36- XVII/102/2010 |  |
| MH996918 | 2009 | Nigeria | Anas platyrhynchos | domestic duck/Nigeria/BA/BAU-R/07T/N17/700/2009 |  |
| MH996919 | 2009 | Nigeria | turkey | Turkey/Nigeria/JG/DT/30-31T/N18/701/2009 |  |
| MH996923 | 2009 | Nigeria | eagle | Wild bird Eagle/Nigeria/VRD09/546/N4/690/2009 |  |
| MH996925 | 2009 | Nigeria | Gallus gallus | chicken/Nigeria/BA/TFB/14C/N38/711/2009 |  |
| MH996926 | 2009 | Nigeria | Gallus gallus | chicken/Nigeria/BO/MMC/AGN/06-07T/N42/712/2009 |  |
| MH996927 | 2009 | Nigeria | Gallus gallus | chicken/Nigeria/VRD09/340/N50/721/2009 |  |
| MH996930 | 2009 | Nigeria | Gallus gallus | local chicken/Nigeria/JN/469-XIV/N44/892/2009 |  |
| MH996931 | 2009 | Nigeria | guinea fowl | Guinea fowl/Nigeria/KT/MA/5-6C/N7/693/2009 |  |
| MH996932 | 2002/2003 | Nigeria | vulture | Vulture/Nigeria/PL038-XVII/N47/895/2002-2003 |  |
| MH996933 | 2009 | Nigeria | Gallus gallus | local chicken/Nigeria/LTS/11T/N38/936/2009 |  |
| MH996934 | 2009 | Nigeria | duck | duck/Nigeria/YB/GSHI/07T-XIV/N5/691/2009 |  |
| MH996935 | 2009 | Nigeria | duck | duck/Nigeria/YB/GSHI/07T-XVII/N5/691/2009 |  |
| MH996937 | 2009 | Nigeria | Anas platyrhynchos | domestic duck/Nigeria/KT/KNK/01T/N13/697/2009 |  |
| MH996939 | 2009 | Nigeria | Anas platyrhynchos | domestic duck/Nigeria/JG/SH/47C-XVII/N15/698/2009 |  |
| MH996940 | 2009 | Nigeria | Anas platyrhynchos | domestic duck/Nigeria/NS/KR/60-61C/N16/699/2009 |  |
| MH996941 | 2009 | Nigeria | Gallus gallus | chicken/Nigeria/NS/KF/06-09C/N46/704/2009 |  |
| MH996942 | 2009 | Nigeria | Anas platyrhynchos | domestic duck/Nigeria/GM/GMM/17-18T/N14/707/2009 |  |
| MH996945 | 2009 | Nigeria | Gallus gallus | chicken/Nigeria/VRD09/025/N21/723/2009 |  |
| MH996946 | 2009 | Nigeria | Gallus gallus | chicken/Nigeria/KT/JBY/09T/N40/733/2009 |  |
| MH996954 | 23-Jul-10 | Nigeria | Gallus gallus | chicken/Nigeria/Dundubus/VRD255/62/2010 |  |
| MH996955 | 23-Aug-13 | Nigeria | Gallus gallus | chicken/Nigeria/VRD158A/70/2013 |  |
| MH996956 | 15-Jun-06 | Nigeria | Gallus gallus | chicken/Nigeria/Kaduna/VRD401/17/2006 |  |
| MH996957 | 14-Feb-07 | Nigeria | Gallus gallus | chicken/Nigeria/Kurmi/VRD033/23/2007 |  |
| MH996958 | 28-Sep-09 | Nigeria | Gallus gallus | chicken/Nigeria/Kwara/VRD415/57/2009 |  |
| MH996959 | 22-Aug-13 | Nigeria | Gallus gallus | chicken/Nigeria/Vom/VRD216/73/2013 |  |
| MH996960 | 12-Dec-08 | Nigeria | Gallus gallus | chicken/Nigeria/Katsina/KT-CH-C18-22/74/2008 |  |
| MH996961 | 15-May-02 | Nigeria | Gallus gallus | chicken/Nigeria/Jos/VRD234/7/2002 |  |
| MH996962 | 2005 | Nigeria | Gallus gallus | chicken/Nigeria/Plateau/VRD26/13/2005 |  |
| MH996963 | 5-Nov-11 | Nigeria | Gallus gallus | chicken/Nigeria/VRD21/63/2011 |  |
| MH996964 | 2012 | Nigeria | Gallus gallus | chicken/Nigeria/VRD64/66/2012 |  |
| MH996966 | 3-Mar-10 | Nigeria | Gallus gallus | chicken/Nigeria/Azare/BA-AZR-C6-7/96/2010 |  |
| MH996967 | 2004 | Nigeria | Gallus gallus | chicken/Nigeria/VRD30/10/2004 |  |
| MH996969 | 2005 | Nigeria | Gallus gallus | chicken/Nigeria/Bauchi/VRD154/14/2005 |  |
| MH996970 | 2-Nov-06 | Nigeria | Gallus gallus | chicken/Nigeria/Zaria/VRD646/19/2006 |  |
| MH996971 | 2-Nov-06 | Nigeria | Gallus gallus | chicken/Nigeria/T/Wada/VRD647/20/2006 |  |
| MH996972 | 2007 | Nigeria | Gallus gallus | chicken/Nigeria/Kaduna/VRD122/28/2007 |  |
| MH996973 | 15-Mar-07 | Nigeria | Gallus gallus | chicken/Nigeria/Katsina/VRD221/40/2007 |  |
| MH996974 | 17-Apr-07 | Nigeria | Gallus gallus | chicken/Nigeria/Fune/VRD284/44/2007 |  |
| MH996975 | 17-Apr-07 | Nigeria | Gallus gallus | chicken/Nigeria/P/Harcourt/VRD289/46/2007 |  |
| MH996976 | 11-Jun-08 | Nigeria | Gallus gallus | chicken/Nigeria/Gombe/VRD316/56/2008 |  |
| MH996977 | 17-Mar-10 | Nigeria | Gallus gallus | chicken/Nigeria/Jigawa/JG-BR-T15/101/2010 |  |
| MN043960 | 2014 | Nigeria | chicken | VRD14.12/2014 |  |
| MN342233 | 1991 | Viet Nam | Avian | VN-91 |  |
| MT362718 | Apr-18 | India | Commercial poultry | Amar |  |
| MT668591 | 1998 | China | goose | goose/Guangdong/FS25/1998 | cloacal |
| MT543153 | 18-Apr-19 | Niger | Gallus gallus | 89 |  |
| MT543159 | 26-Apr-19 | Niger | Gallus gallus | 255 |  |
| MT543160 | 27-Apr-19 | Niger | Gallus gallus | 261 |  |
| MT120188 | 2010 | China: Yunnan | chicken | 7 | allantoic fluid |
| MT120190 | 2010 | China: Yunnan | chicken | 9 | allantoic fluid |
| MT120192 | 2010 | China: Yunnan | chicken | 11 | allantoic fluid |
| LC650527 | 1985 | Japan | Phasianus versicolor | APMV1/pheasant/Japan/Gunma/85 |  |
| LC650529 | 1985 | Japan | Gallus gallus domesticus | APMV1/chicken/Japan/Niigata/85 |  |
| LC650530 | 1985 | Japan | Gallus gallus domesticus | APMV1/chicken/Japan/Saitama/85 |  |
| LC650531 | 1985 | Japan | Gallus gallus domesticus | APMV1/chicken/Japan/Shizuoka/85 |  |
| LC650534 | 1986 | Japan | Gallus gallus domesticus | APMV1/chicken/Japan/Chiba/86 |  |
| LC650535 | 1987 | Japan | Gallus gallus domesticus | APMV1/chicken/Japan/Chiba/87 |  |
| LC650537 | 1989 | Japan | Gallus gallus domesticus | APMV1/chicken/Japan/Nara/89 |  |
| LC650538 | 1989 | Japan | Gallus gallus domesticus | APMV1/chicken/Japan/Niigata/89 |  |
| LC650547 | 1997 | Japan | Phasianus versicolor | APMV1/pheasant/Japan/Ibaraki/97 |  |
| LC650548 | 1999 | Japan | Gallus gallus domesticus | APMV1/chicken/Japan/Ibaraki-1/99 |  |
| LC650550 | 2000 | Japan | Gallus gallus domesticus | APMV1/chicken/Japan/Ibaraki/2000 |  |
| LC650557 | 2002 | Japan | Gallus gallus domesticus | APMV1/chicken/Japan/Okayama-1/2002 |  |
| MZ306211 | 20-Mar-00 | China | pigeon | pigeon/China/GXD12/2000 |  |
| MZ306213 | Feb-00 | China | chicken | chicken/China/GXD23/2000 |  |
| OK491971 | 20-Mar-20 | Nigeria |  | KN14 | chicken offal |
| OK491972 | 25-Mar-20 | Nigeria |  | KN36 | chicken offal |
| OK491973 | 30-Mar-20 | Nigeria |  | KN48 | chicken offal |
| OK491974 | 31-Mar-20 | Nigeria |  | KN55 | chicken offal |
| OK491975 | 31-Mar-20 | Nigeria |  | KN56 | chicken offal |
| OK491976 | 2-Apr-20 | Nigeria |  | KN71 | chicken offal |
| OK491977 | 3-Apr-20 | Nigeria |  | KN75 | chicken offal |
| ON645960 | 2005 | North Macedonia | chicken | AOAV-1/chicken/Macedonia/070/2005 |  |
| ON645961 | 2004 | North Macedonia | chicken | AOAV-1/chicken/Macedonia/080/2004 |  |
| OM135588 | 2006 | China | Gallus gallus | NDV/Chicken/Jiangsu/17/2006 |  |
| OP818743 | 1995 | Korea |  | Kr-279/95 |  |
| OP818780 | 2002 | China |  | ch-501/02 |  |
| OP818781 | 2001 | China |  | ch-77/01 |  |
| OP818782 | 1984 | Korea |  | Kr_D/84 |  |
| OP818788 | 2000 | Korea |  | Kr_420/00 |  |
| OP818789 | 2000 | Korea |  | Kr_009/00 |  |
| OP818791 | 2000 | Korea |  | Kr_352/00 |  |
| OP818792 | 2000 | Korea |  | Kr_400/00 |  |
| OP818794 | 2000 | Korea |  | 60 |  |
| OP818796 | 2000 | Korea |  | 707 |  |
| OP818799 | 2001 | Korea |  | 1194 |  |
| OP818800 | 2002 | Korea |  | 2188 |  |
| OP818803 | 2002 | Korea |  | 2413 |  |
| OP818804 | 2002 | Korea |  | 2415 |  |
| OP818805 | 2002 | Korea |  | 2222 |  |
| OP818806 | 2002 | Korea |  | 2239 |  |
| OP818807 | 2002 | Korea |  | 2254 |  |
| OP818809 | 2002 | Korea |  | 2262 |  |
| OP818844 | 1995 | Korea |  | Kr-146/95 |  |
| OQ920548 | 2019 | Nigeria | Chicken | APMV-1/Chicken/Nigeria/F0101C8S/2019 |  |
| OQ920549 | 2019 | Nigeria | Chicken | APMV-1/Chicken/Nigeria/F0201C8P/2019 |  |
| OQ920550 | 2019 | Nigeria | Chicken | APMV-1/Chicken/Nigeria/F0301C8P/2019 |  |
| OQ920551 | 2019 | Nigeria | Chicken | APMV-1/Chicken/Nigeria/F1402C1OS/2019 |  |
| OQ920552 | 2019 | Nigeria | Chicken | APMV-1/Chicken/Nigeria/F1801C8S/2019 |  |
| OQ920553 | 2019 | Nigeria | Chicken | APMV-1/Chicken/Nigeria/F0601C8LU/2019 |  |
| OR367436 | 2010 | Pakistan | Gallus gallus | AoAV-1/UVAS-Pak/Ck/2010 | trachea |
| PP738481 | 30-Apr-13 | Kazakhstan | Gallus gallus | NDV/PMV-1/chicken/Astana/49/98 |  |
| PP788551 | 23-Jun-05 | Peru | Gallus gallus | Chicken/Chancay/Peru/050566/2005 |  |
| PP788552 | 13-Apr-11 | Peru | Gallus gallus | Chicken/Ancash/Peru/110288/2011 |  |
| PP788554 | 26-Aug-15 | Peru | Gallus gallus | Chicken/Lima/Peru/150795/2015 |  |
| PQ043263 | 2023 | China | Pigeon | Pigeon/China/Hebei/01/2023 | spleen |
| PV339969 | 30-Nov-23 | Nigeria | Chicken | chicken/Borno/Nigeria/SJ15/2023 | trachea, spleen |
| PX095534 | 22-Dec-23 | Nigeria | Chicken | SJ55 | swab |
| PX095535 | 24-Jan-24 | Nigeria | Chicken | SJ106 | tissues |
| PX095536 | 15-May-24 | Nigeria | Chicken | SJ117 | tissues |
| PX095537 | 15-May-24 | Nigeria | Chicken | SJ124 | tissues |
| PX095538 | 18-May-24 | Nigeria | Chicken | SJ126 | tissues |
| PX095539 | 18-May-24 | Nigeria | Chicken | SJ129 | tissues |
| PX095540 | 18-May-24 | Nigeria | Chicken | SJ130 | tissues |

Suppl. Table 2: The details of the NDV full-length F gene sequences of the West African isolates used for the Nextstrain analysis

| Accession | Host | Date | Country-Location | Coordinates |
| --- | --- | --- | --- | --- |
| FJ772446 | Aves | 2006.1.1 | Nigeria-Abuja | 9.054171, 7.497098 |
| FJ772449 | Aves | 2006.1.1 | Nigeria-Abuja | 9.054171, 7.497098 |
| FJ772452 | Gallus gallus | 2006.1.1 | Niger-Niger | 13.520767, 2.103578 |
| FJ772455 | Aves | 2006.1.1 | Mauritania-Mauritania | 17.951949, -14.939584 |
| FJ772458 | Gallus gallus | 2008.1.1 | Burkina_Faso-Ouagadougou | 12.333313, -1.535381 |
| FJ772469 | Gallus gallus | 2008.1.1 | Niger-Niger | 13.520767, 2.103578 |
| FJ772472 | Gallus gallus | 2008.1.1 | Niger-Niger | 13.520767, 2.103578 |
| FJ772481 | Gallus gallus | 2008.1.1 | Niger-Niger | 13.520767, 2.103578 |
| FJ772486 | Aves | 2008.1.1 | Nigeria-Abuja | 9.054171, 7.497098 |
| JF966385 | Gallus gallus | 2008.1.1 | Mali-Mali | 12.599008, -7.984653 |
| JF966386 | Gallus gallus | 2007.1.1 | Mali-Mali | 12.599008, -7.984653 |
| JF966387 | Gallus gallus | 2009.1.1 | Mali-Mali | 12.599008, -7.984653 |
| JF966388 | Gallus gallus | 2008.1.1 | Mali-Mali | 12.599008, -7.984653 |
| JF966389 | Numididae | 2007.1.1 | Mali-Mali | 12.599008, -7.984653 |
| JN872165 | Gallus gallus | 2006.1.1 | Niger-Niger | 13.520767, 2.103578 |
| JQ039386 | Gallus gallus | 2008.1.1 | Nigeria-Plateau-Mangu | 9.491270, 9.141894 |
| JQ039390 | Gallus gallus | 2007.1.1 | Nigeria-Katsina-Batagarawa | 12.906489, 7.601308 |
| JQ039393 | Gallus gallus | 2007.1.1 | Nigeria-Sokoto-Tambara | 13.149739, 5.409383 |
| JQ039394 | Gallus gallus | 2007.1.1 | Nigeria-Jigawa-Kafin Hausa | 12.237968, 9.901891 |
| JX390609 | Gallus gallus | 2009.1.1 | Togo-Togo | 7.195754, 1.079138 |
| JX518885 | Gallus gallus | 2010.1.1 | Mali-Bamako | 12.599008, -7.984653 |
| JX518886 | Gallus gallus | 2010.1.1 | Mali-Bamako | 12.599008, -7.984653 |
| JX546245 | Gallus gallus | 2009.1.1 | Benin-Benin | 9.958131, 2.234052 |
| JX546247 | Gallus gallus | 2009.1.1 | Benin-Benin | 9.958131, 2.234052 |
| KC568204 | Columba livia | 2009.7.1 | Nigeria-Zamfara-Kaura Namoda | 12.592482, 6.586530 |
| KC568205 | Numida meleagris | 2009.8.1 | Nigeria-Kaduna | 10.510055, 7.434648 |
| KC568206 | Numida meleagris | 2009.8.1 | Nigeria-Jigawa-Kazaure | 12.647088, 8.410594 |
| KC568208 | Gallus gallus | 2009.6.1 | Nigeria-Gombe | 10.301150, 11.159706 |
| KF442614 | Gallus gallus | 2006.1.1 | Nigeria-Oyo-Ibadan | 7.410061, 3.906429 |
| KT948996 | Anas platyrhynchos | 2009.6.1 | Nigeria-Kogi-Lokoja | 7.802383, 6.732371 |
| KY171992 | Gallus gallus | 2009.1.1 | Nigeria-Plateau-Jos North | 9.940700, 8.886983 |
| MH092808 | Meleagris gallopavo | 2009.1.1 | Nigeria-Plateau-Jos North | 9.940700, 8.886983 |
| MH092809 | Anatidae | 2009.1.1 | Nigeria- Plateau -Langtang South | 8.626489, 9.815322 |
| MH092810 | Anatidae | 2009.1.1 | Nigeria-Plateau-Kanam | 9.561687, 9.951729 |
| MH092816 | Gallus gallus | 2009.1.1 | Nigeria-Plateau-Wase | 9.096581, 9.959832 |
| MH092817 | Gallus gallus | 2009.1.1 | Nigeria-Plateau-Wase | 9.096581, 9.959832 |
| MH092821 | Gallus gallus | 2009.1.1 | Nigeria- Plateau-Jos North | 9.940700, 8.886983 |
| MH392225 | Gallus gallus | 2009.1.1 | Nigeria-Kaduna | 10.515302, 7.434598 |
| MH392227 | Gallus gallus | 2009.1.1 | Nigeria-Ota Ogun | 6.688913, 3.235293 |
| MH996907 | Gallus gallus | 2009.1.1 | Nigeria-Plateau-Jos North | 9.940700, 8.886983 |
| MH996918 | Anas platyrhynchos | 2009.1.1 | Nigeria-Bauchi | 10.317031, 9.848775 |
| MH996919 | Meleagris gallopavo | 2009.1.1 | Nigeria-Jigawa-Dutse | 11.717662, 9.358536 |
| MH996923 | Accipitridae | 2009.1.1 | Nigeria-Taraba | 8.870469, 11.389513 |
| MH996925 | Gallus gallus | 2009.1.1 | Nigeria-Bauchi | 10.317031, 9.848775 |
| MH996926 | Gallus gallus | 2009.1.1 | Nigeria-Borno-Maiduguri | 11.835608, 13.152259 |
| MH996927 | Gallus gallus | 2009.1.1 | Nigeria-Kebbi | 12.409904, 4.172129 |
| MH996930 | Gallus gallus | 2009.1.1 | Nigeria- Plateau-Jos North | 9.945985, 8.874684 |
| MH996931 | Numididae | 2009.1.1 | Nigeria-Katsina-Mai'Adua | 13.147104, 8.227065 |
| MH996932 | Gyps fulvus | 2003.1.1 | Nigeria- Plateau-Langtang South | 8.626489, 9.815322 |
| MH996933 | Gallus gallus | 2003.1.1 | Nigeria- Plateau -Langtang South | 8.626489, 9.815322 |
| MH996934 | Anatidae | 2009.1.1 | Nigeria-Yobe-Gashua | 12.872327, 11.031396 |
| MH996935 | Anatidae | 2009.1.1 | Nigeria-Yobe-Gashua | 12.872327, 11.031396 |
| MH996937 | Anas platyrhynchos | 2009.1.1 | Nigeria-Katsina-Kankia | 12.446841, 7.793827 |
| MH996939 | Anas platyrhynchos | 2009.1.1 | Nigeria-Jigawa | 11.717662, 9.358536 |
| MH996940 | Anas platyrhynchos | 2009.1.1 | Nigeria-Nasarawa-Karu | 8.977111, 7.728063 |
| MH996941 | Gallus gallus | 2009.1.1 | Nigeria-Nasarawa-Keffi | 8.850520, 7.875556 |
| MH996942 | Anas platyrhynchos | 2009.1.1 | Nigeria-Gombe | 10.296302, 11.174248 |
| MH996945 | Gallus gallus | 2009.1.1 | Nigeria-Kano | 12.006894, 8.495918 |
| MH996946 | Gallus gallus | 2009.1.1 | Nigeria-Katsina-Jibiya | 13.103186, 7.215463 |
| MH996954 | Gallus gallus | 2010.7.23 | Nigeria-Bauchi-Shira-Dundubus | 11.459999, 10.043052 |
| MH996956 | Gallus gallus | 2006.6.15 | Nigeria-Kaduna | 10.515302, 7.434598 |
| MH996957 | Gallus gallus | 2007.2.14 | Nigeria-Taraba-Kurmi | 7.861679, 10.984013 |
| MH996958 | Gallus gallus | 2009.9.28 | Nigeria-Kwara-Ilorin | 8.471772, 4.577613 |
| MH996960 | Gallus gallus | 2008.12.12 | Nigeria-Katsina-Charanchi | 12.666236, 7.735263 |
| MH996961 | Gallus gallus | 2002.5.15 | Nigeria- Plateau-Jos North | 9.945985, 8.874684 |
| MH996962 | Gallus gallus | 2005.1.1 | Nigeria-Plateau-Jos North | 9.945985, 8.874684 |
| MH996966 | Gallus gallus | 2012.1.1 | Nigeria-Bauchi-Azare | 11.677348, 10.197853 |
| MH996969 | Gallus gallus | 2005.1.1 | Nigeria-Bauchi | 10.312808, 9.843282 |
| MH996970 | Gallus gallus | 2006.11.2 | Nigeria-Kaduna-Zaria | 11.127723, 7.720816 |
| MH996971 | Gallus gallus | 2006.11.2 | Nigeria-Kaduna-Tudun Wada | 10.510914, 7.408848 |
| MH996972 | Gallus gallus | 2007.1.1 | Nigeria-Kaduna | 10.515302, 7.434598 |
| MH996973 | Gallus gallus | 2007.3.15 | Nigeria-Katsina | 12.994625, 7.617952 |
| MH996974 | Gallus gallus | 2007.4.17 | Nigeria-Yobe-Fune | 11.684180, 11.327826 |
| MH996975 | Gallus gallus | 2007.4.17 | Nigeria-Rivers-Port Harcourt | 4.831090, 7.020928 |
| MH996976 | Gallus gallus | 2008.6.11 | Nigeria-Gombe | 10.296302, 11.174248 |
| MH996977 | Gallus gallus | 2010.3.17 | Nigeria-Jigawa-Biriniwa | 12.790254, 10.235481 |
| MH996980 | Gallus gallus | 2013.5.30 | Nigeria-Vwang, Jos South | 9.712636, 8.739119 |
| MH996981 | Gallus gallus | 2015.4.1 | Nigeria-Imo-Owerri-west | 5.523076, 6.949630 |
| MH996982 | Gallus gallus | 2015.4.1 | Nigeria-Imo-Owerri-west | 5.523076, 6.949630 |
| MH996984 | Gallus gallus | 2011.8.18 | Nigeria-Katsina | 12.994625, 7.617952 |
| MH996986 | Gallus gallus | 2009.12.21 | Nigeria-Adamawa | 9.201153, 12.483582 |
| MH996987 | Gallus gallus | 2009.12.21 | Nigeria-Kogi | 7.802383, 6.732371 |
| MH996988 | Gallus gallus | 2010.11.4 | Nigeria-Nasarawa-Keffi | 8.850520, 7.875556 |
| MH996989 | Gallus gallus | 2010.8.28 | Nigeria-Jigawa-Dutse | 11.717662, 9.358536 |
| MH996990 | Gallus gallus | 2010.8.28 | Nigeria-Jigawa-Dutse | 11.717662, 9.358536 |
| MT543153 | Gallus gallus | 2019.4.18 | Niger-Niger-Maradi | 13.496448, 7.101691 |
| MT543159 | Gallus gallus | 2019.4.26 | Niger-Niger-Tillaberi | 14.206920, 1.456365 |
| MT543160 | Gallus gallus | 2019.4.27 | Niger-Niger-Tillaberi | 14.206920, 1.456365 |
| OK491971 | Gallus gallus | 2020.3.20 | Nigeria-Kano | 12.006894, 8.495918 |
| OK491972 | Gallus gallus | 2020.3.25 | Nigeria-Kano | 12.006894, 8.495918 |
| OK491973 | Gallus gallus | 2020.3.30 | Nigeria-Kano | 12.006894, 8.495918 |
| OK491974 | Gallus gallus | 2020.3.31 | Nigeria-Kano | 12.006894, 8.495918 |
| OK491975 | Gallus gallus | 2020.3.31 | Nigeria-Kano | 12.006894, 8.495918 |
| OK491976 | Gallus gallus | 2020.4.2 | Nigeria-Kano | 12.006894, 8.495918 |
| OK491977 | Gallus gallus | 2020.4.3 | Nigeria-Kano | 12.006894, 8.495918 |
| OQ920548 | Gallus gallus | 2019.1.1 | Nigeria-Kano | 12.006894, 8.495918 |
| OQ920549 | Gallus gallus | 2019.1.1 | Nigeria-Kano | 12.006894, 8.495918 |
| OQ920550 | Gallus gallus | 2019.1.1 | Nigeria-Kano | 12.006894, 8.495918 |
| OQ920551 | Gallus gallus | 2019.1.1 | Nigeria-Abuja | 9.054171, 7.497098 |
| OQ920552 | Gallus gallus | 2019.1.1 | Nigeria-Abuja | 9.054171, 7.497098 |
| OQ920553 | Gallus gallus | 2019.1.1 | Nigeria-Kano | 12.006894, 8.495918 |
| PV339969 | Gallus gallus | 2023.11.30 | Nigeria-Borno-Maiduguri | 11.835608, 13.152259 |
| SJ106 | Gallus gallus | 2024.1.24 | Nigeria-Taraba-Jalingo | 8.894608, 11.359054 |
| SJ117 | Gallus gallus | 2024.5.15 | Nigeria-Borno-Maiduguri | 11.835608, 13.152259 |
| SJ124 | Gallus gallus | 2024.5.15 | Nigeria-Borno-Maiduguri | 11.835608, 13.152259 |
| SJ126 | Gallus gallus | 2024.5.18 | Nigeria-Yobe-Damaturu | 11.7382305, 11.9322155 |
| SJ129 | Gallus gallus | 2024.5.18 | Nigeria-Yobe-Damaturu | 11.7382305, 11.9322155 |
| SJ130 | Gallus gallus | 2024.5.18 | Nigeria-Yobe-Damaturu | 11.7382305, 11.9322155 |
| SJ55 | Gallus gallus | 2023.12.22 | Nigeria-Gombe | 10.301150, 11.159706 |
