# Supplementary material for: Evolution and Spread of Regionally Adapted Newcastle Disease Virus Isolates From Live Bird Markets in Nigeria, 2023–2024
Source: Transbound Emerg Dis. 2026 Jan 24;2026:8829822. doi: 10.1155/tbed/8829822 (PMC12831130; doi:10.1155/tbed/8829822)
Supplement: Supplementary file 2 — Supporting Information 2 Figure S1: Root to tip of the maximum likelihood (ML) tree of the 286 full‐length F gene sequences from this study. Figure S2: The ML tree of 286 full‐length F gene sequences. The seven study samples are marked in red. Figure S3: The ML tree of genotype XIV from Nigeria. The seven study samples are marked in red. Figure S4: The ML tree of genotype XVII from Nigeria. Figure S5: The ML tree of 428 full‐length HN gene sequences. Figure S6: Genotype XIV codon site 115 negative/purifying selection by fast, unconstrained Bayesian AppRoximation (FUBAR) graph showing the posterior distribution over the discretized rate grid. Figure S7: Genotype XIV codon site 516 positive/diversifying selection by FUBAR graph showing the posterior distribution over the discretized rate grid. Figure S8: Genotype XIV codon site 517 neutral by FUBAR graph showing the posterior distribution over the discretized rate grid. Figure S9: Genotype XIV mixed effects model of evolution (MEME) site plot showing episodic diversifying selection. Figure S10: Genotype XIV selection pressure single‐likelihood ancestor counting (SLAC) site graph showing a predominant negative selection. Figure S11: Genotype XVII codon site 114 positive/diversifying selection by FUBAR graph. Figure S12: Genotype XVII MEME site plot showing episodic diversifying selection. Figure S13: Genotype XVII selection pressure SLAC site graph showing a predominant negative selection. [file TBED-2026-8829822-s001.docx]

**SUPPLEMENTARY FILE 2**

**Evolution and Spread of Regionally Adapted Newcastle Disease Virus Isolates from Live Bird Markets in Nigeria, 2023-2024**

Mohammed Usman Sajo^1,2,3,4*^, Dongyeop Lee^5^, Jean Nepomuscene Hakizimana^2^, Augustino Chengula^1^, Abdul-Dahiru El-Yuguda^3^, Dong-Hun Lee^4,5*^, Gerald Misinzo^1,2*^

^1^Department of Microbiology, Parasitology and Biotechnology, College of Veterinary Medicine and Biomedical Sciences, Sokoine University of Agriculture, Morogoro, 67152, Tanzania

^2^OR Tambo Africa Research Chair for Viral Epidemics, SACIDS Foundation for One Health, Sokoine University of Agriculture, Morogoro, 67152, Tanzania

^3^Animal Virus Research Laboratory, Department of Veterinary Microbiology, Faculty of Veterinary Medicine, University of Maiduguri, Maiduguri, 600001, Nigeria

^4^Konkuk University Zoonotic Disease Research Center, College of Veterinary Medicine, Konkuk University, Seoul, 05029, Republic of Korea

^5^Wildlife Health Laboratory, College of Veterinary Medicine, Konkuk University, Seoul, 05029, Republic of Korea

*Corresponding authors; MUS: [sajom@unimaid.edu.ng](mailto:sajom@unimaid.edu.ng), DHL: [donghunlee@konkuk.ac.kr](mailto:donghunlee@konkuk.ac.kr), GM: [gerald.misinzo@sacids.org](mailto:gerald.misinzo@sacids.org)

The responsible corresponding author: Gerald Misinzo, Department of Microbiology, Parasitology and Biotechnology, College of Veterinary Medicine and Biomedical Sciences, Sokoine University of Agriculture, Morogoro, 67152, Tanzania

Supplementary Figures


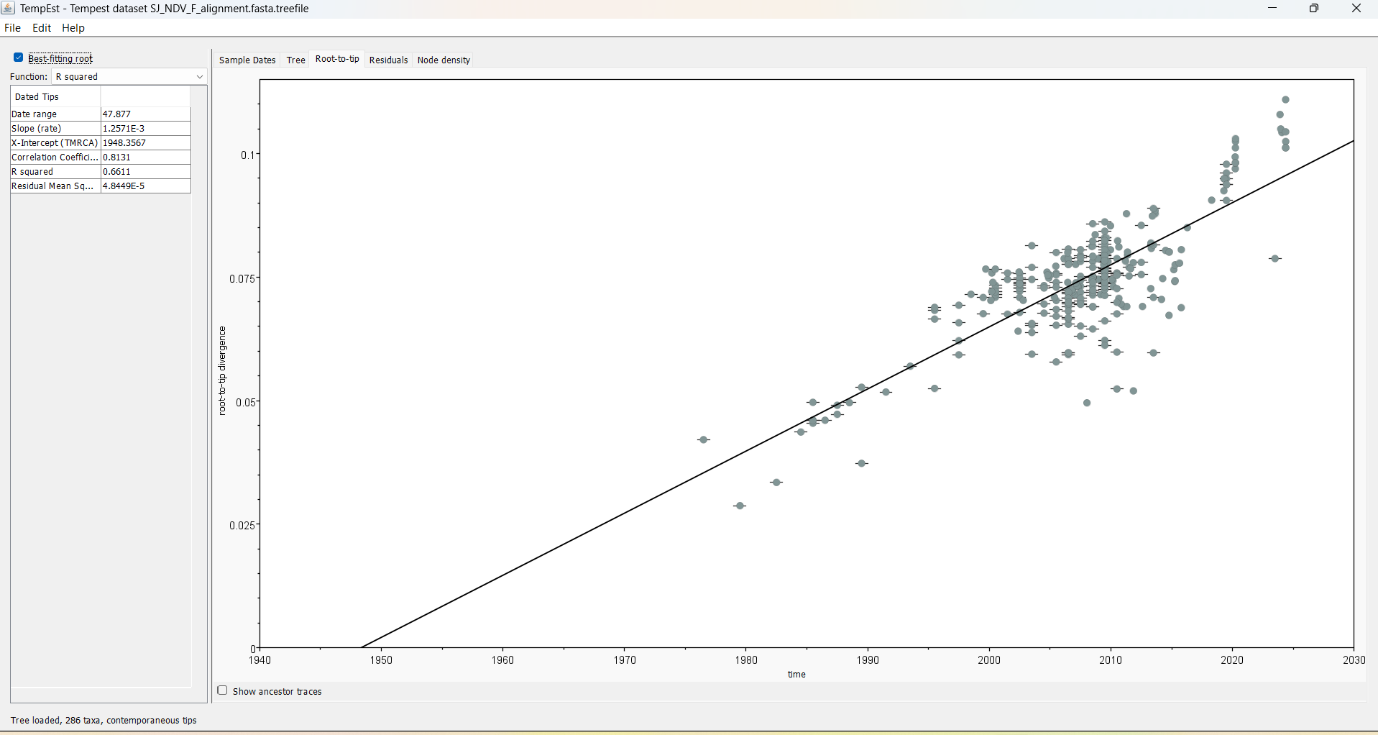


Suppl. Figure 1: Root to tip of the maximum likelihood (ML) tree of the 286 full-length F gene sequences from this study.

Suppl. Figure 2: The ML tree of 286 full-length F gene sequences. The seven study samples are marked in red.

Suppl. Figure 3: The ML tree of Genotype XIV from Nigerian NDV full-length F gene sequences. The seven study samples are marked in red.

Suppl. Figure 4: The ML tree of Genotype XVII from Nigerian NDV full-length F gene sequences.


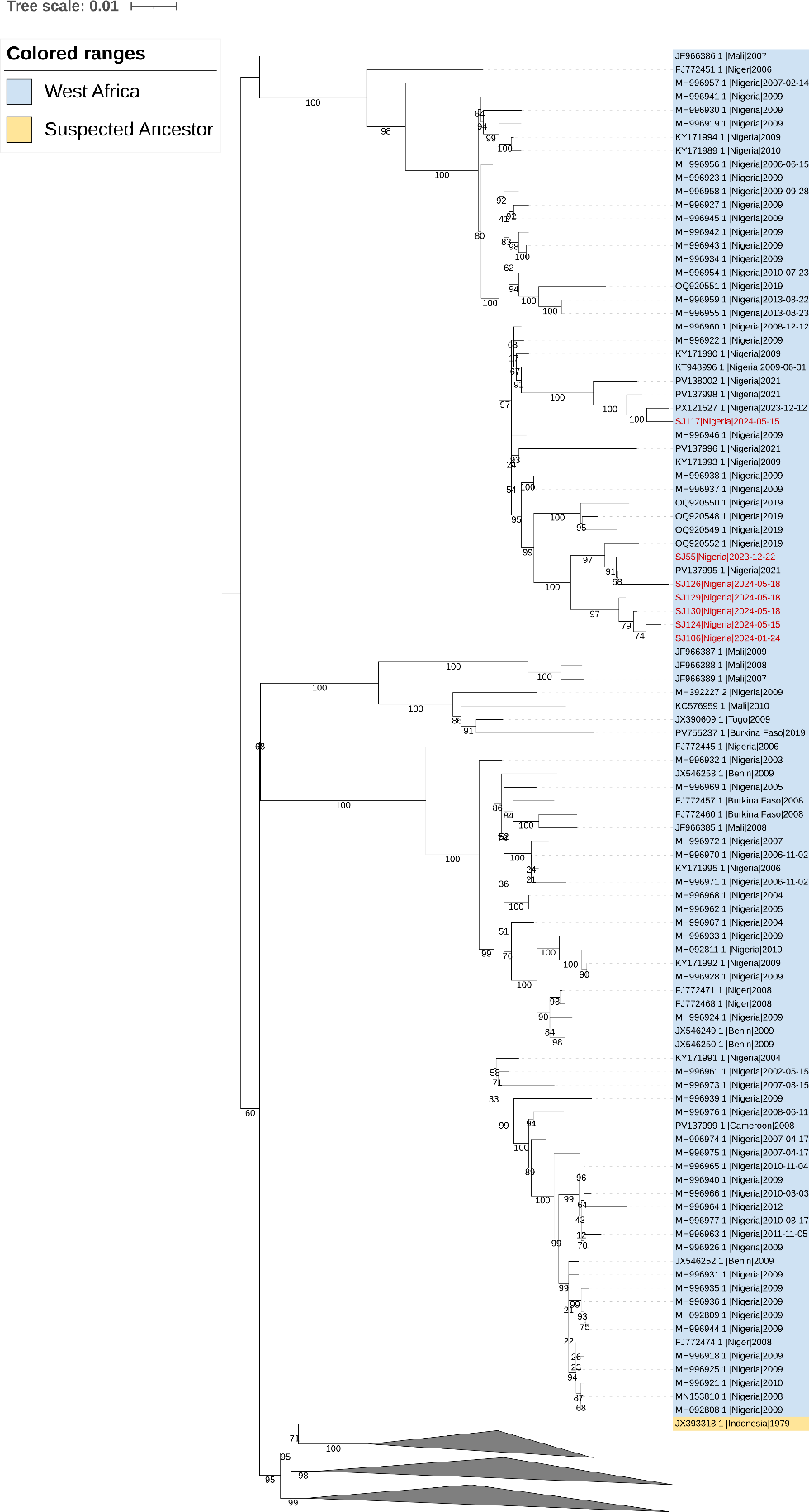


Suppl. Figure 5: The ML tree of 428 full-length HN gene sequences. The seven study samples are marked in red.


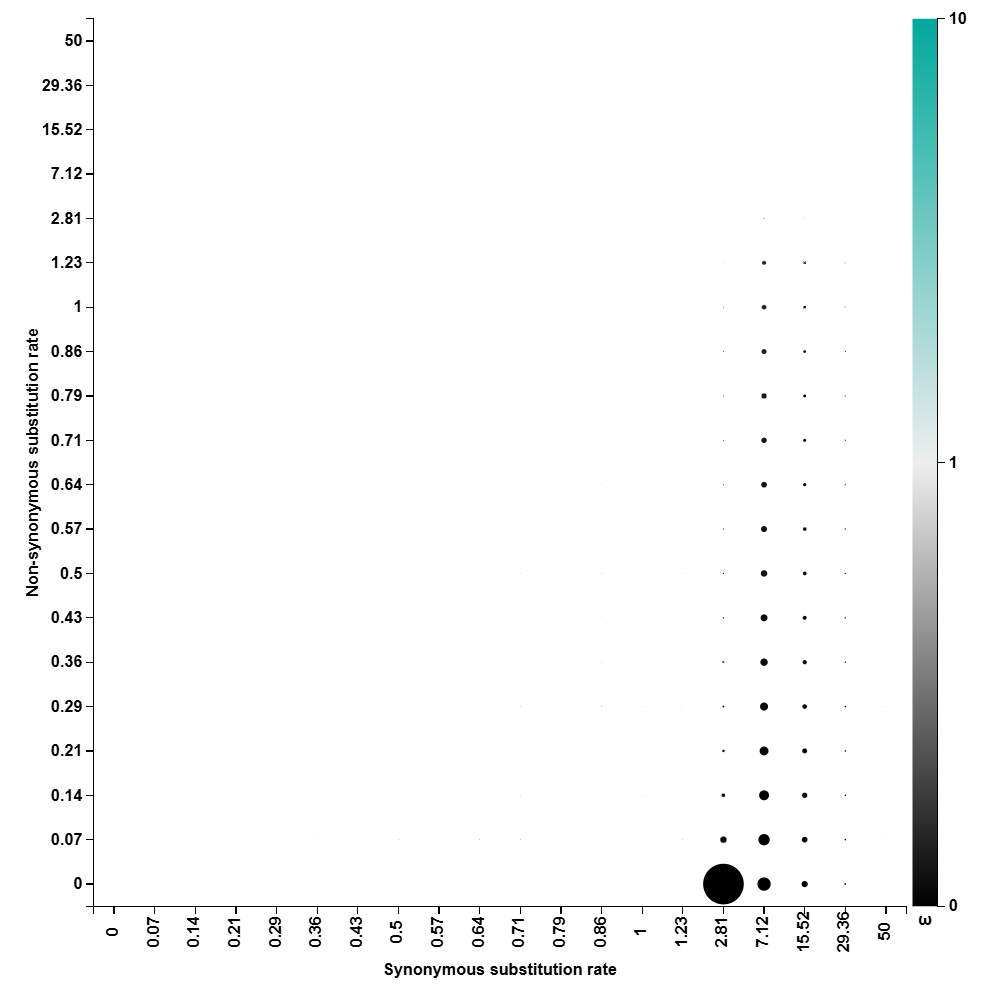


Suppl. Figure 6: Genotype XIV codon site 115 Negative/purifying selection by Fast, Unconstrained Bayesian AppRoximation (FUBAR) Graph showing the posterior distribution over the discretized rate grid. The size of a dot is proportional to the posterior weight allocated to that gridpoint, and the color shows the intensity of selection.


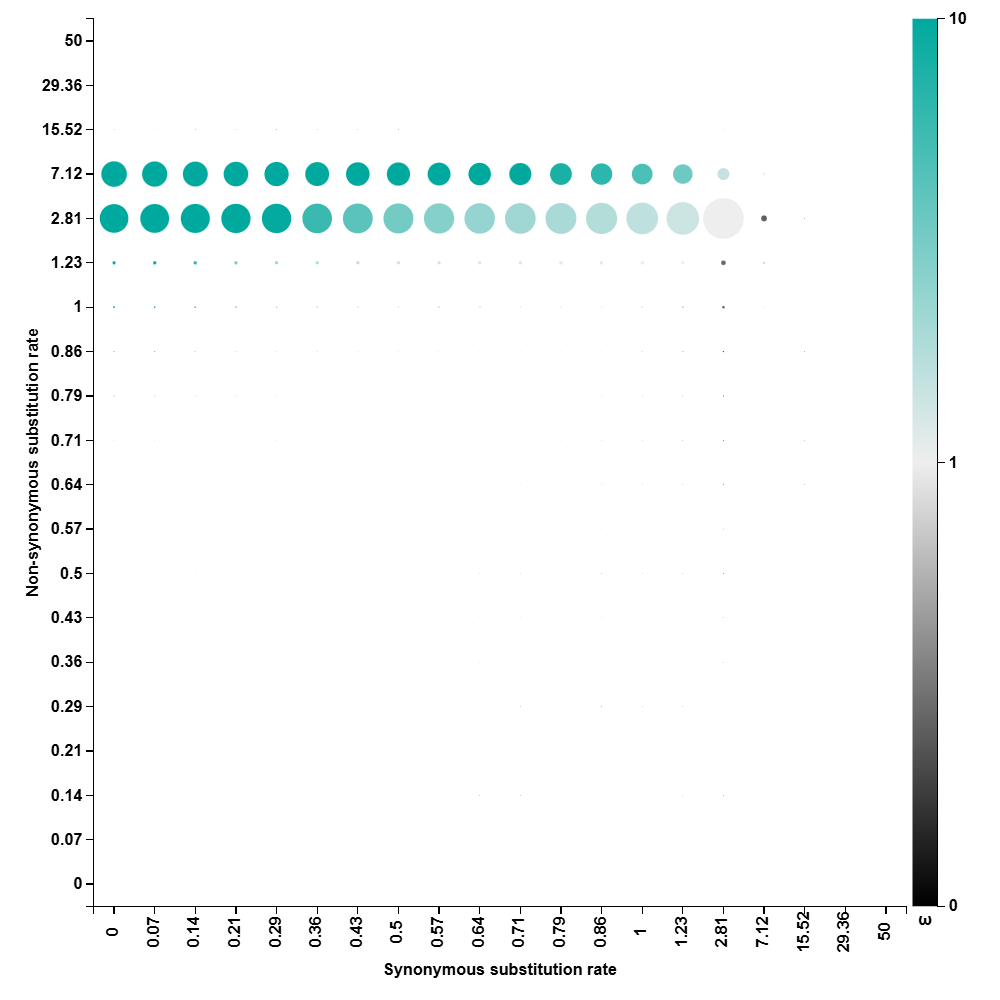


Suppl. Figure 7: Genotype XIV codon site 516 Positive/diversifying selection by FUBAR Graph showing the posterior distribution over the discretized rate grid. The size of a dot is proportional to the posterior weight allocated to that gridpoint, and the color shows the intensity of selection.


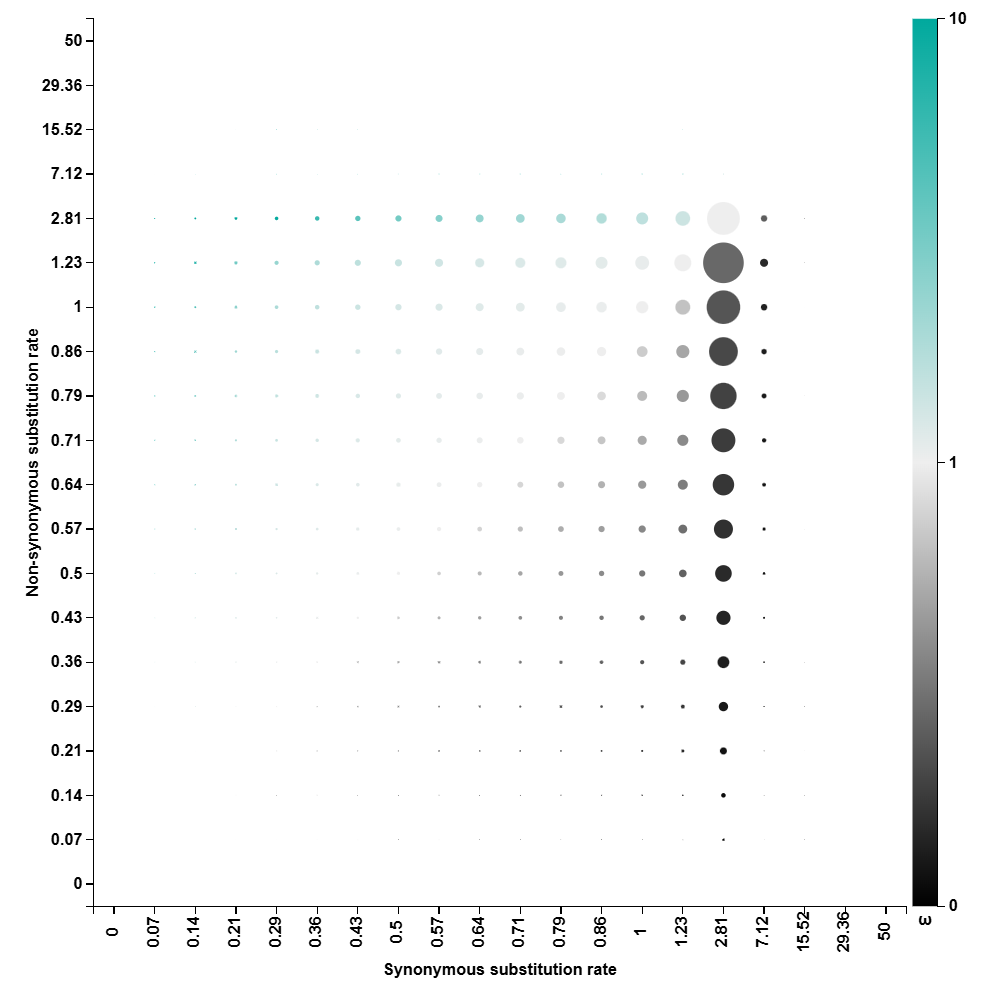


Suppl. Figure 8: Genotype XIV codon site 517 Neutral by FUBAR Graph showing the posterior distribution over the discretized rate grid. The size of a dot is proportional to the posterior weight allocated to that gridpoint, and the color shows the intensity of selection.


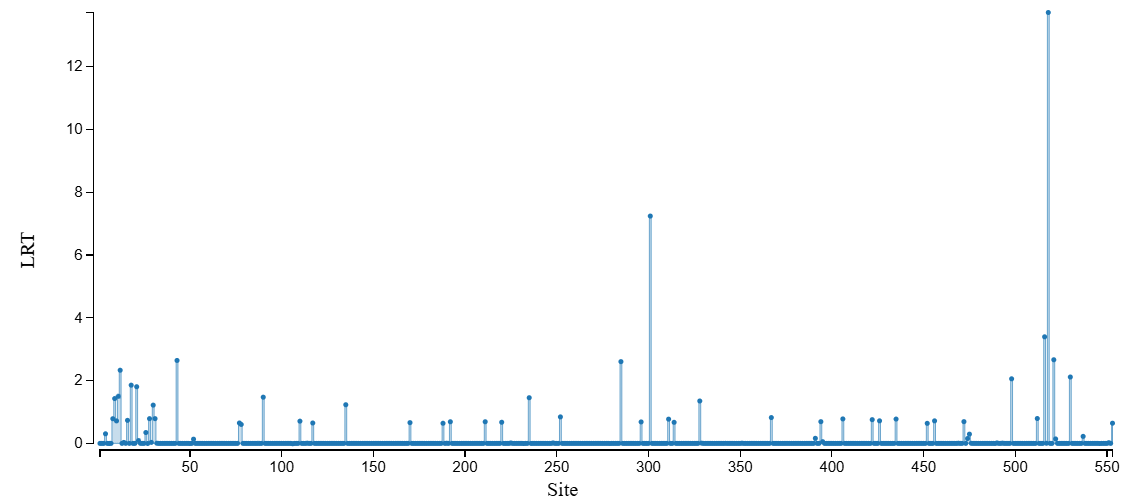


Suppl. Figure 9: Genotype XIV Mixed Effects Model of Evolution (MEME) Site Plot showing episodic diversifying selection at codon site 301, 516 and 518 with LRT value of 7.2, 3.4 and 13.7 respectively (at p < 0.1).


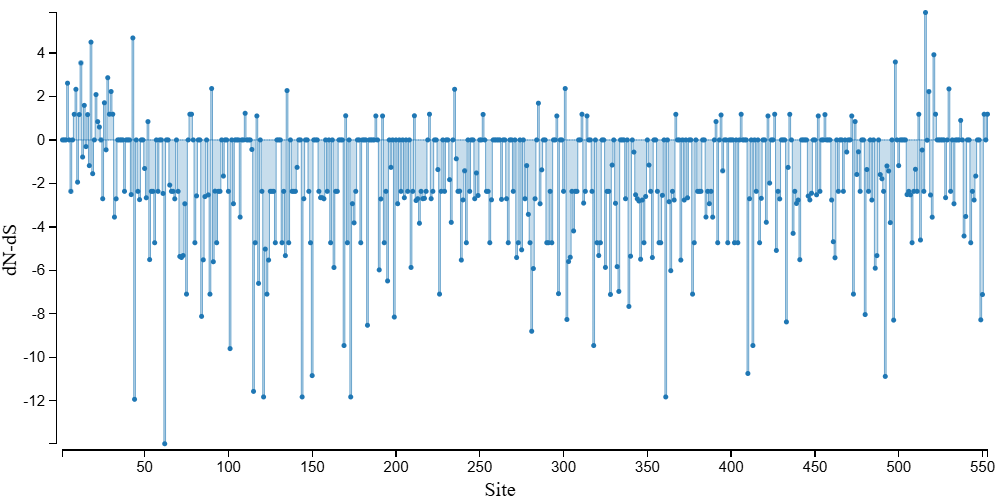


Suppl. Figure 10: Genotype XIV Selection Pressure Single-Likelihood Ancestor Counting (SLAC) Site Graph showing a predominant negative selection (at p < 0.1).


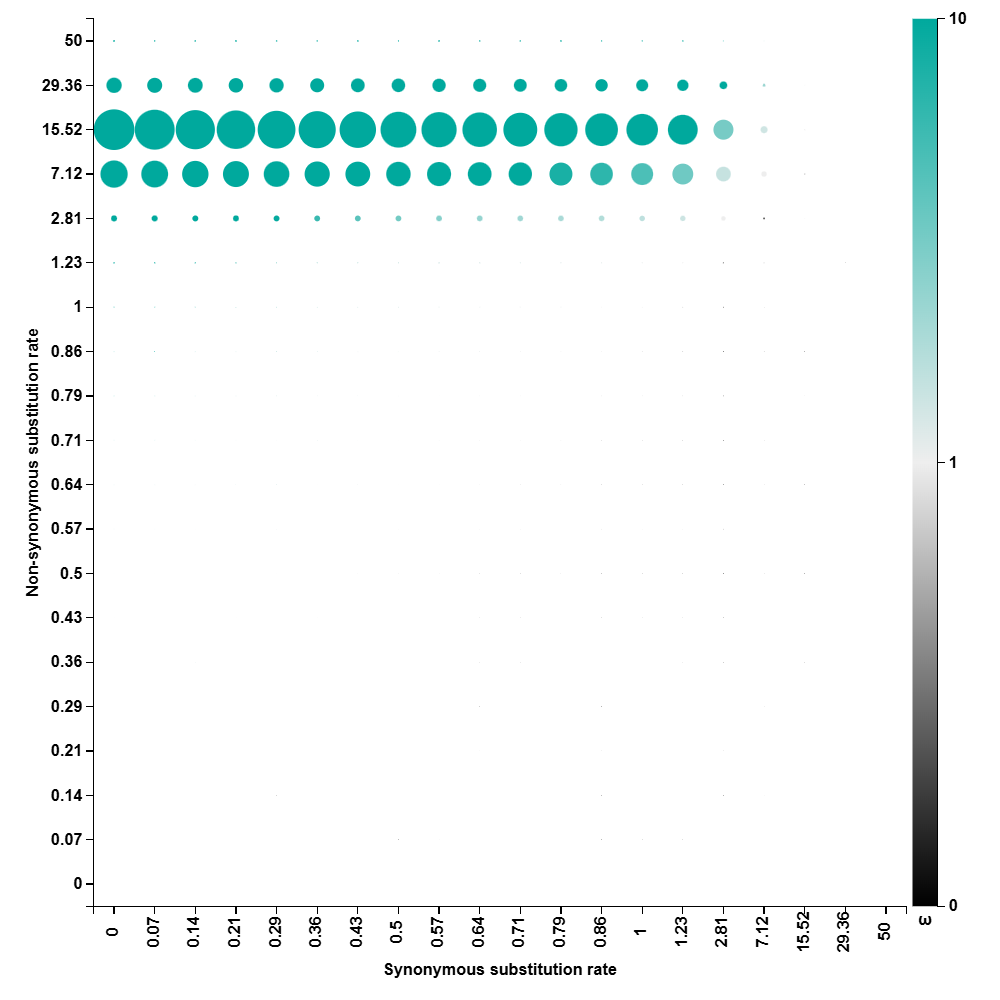


Suppl. Figure 11: Genotype XVII codon site 114 Positive/diversifying selection by FUBAR Graph showing the posterior distribution over the discretized rate grid. The size of a dot is proportional to the posterior weight allocated to that gridpoint, and the color shows the intensity of selection.


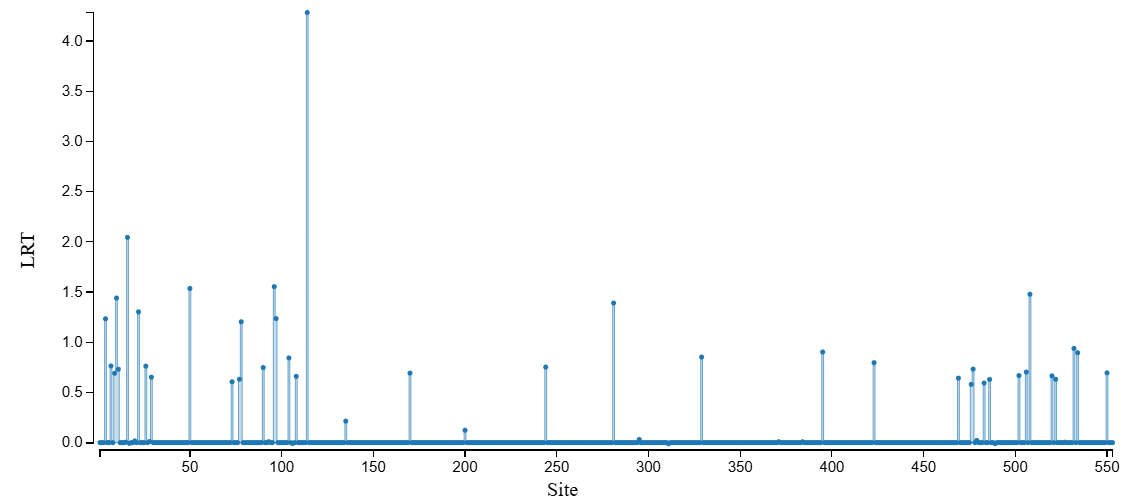


Suppl. Figure 12: Genotype XVII MEME Site Plot showing episodic diversifying selection at codon site 114 with LRT value of 4.3 (at p < 0.1).


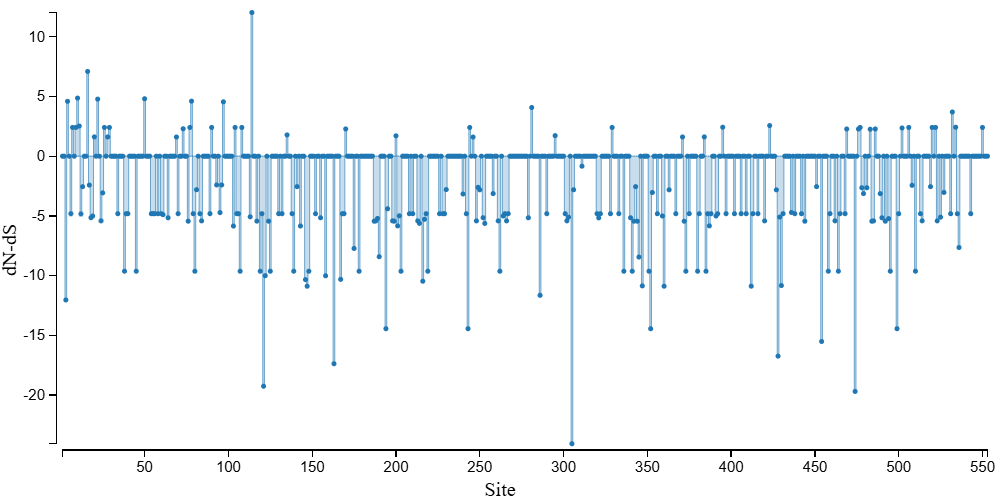


Suppl. Figure 13: Genotype XVII Selection Pressure SLAC Site Graph showing a predominant negative selection (at p < 0.1).
